# A Pump‐Free, Hydraulic‐Amplification Oscillatory Microfluidic Device for Continuous Particle and Cell Manipulation

**DOI:** 10.1002/advs.202507041

**Published:** 2025-05-23

**Authors:** Yong Liu, Mingyi Liang, Shanshan Xu, Sheng Yan

**Affiliations:** ^1^ Institute for Advanced Study Shenzhen University Shenzhen 518060 China; ^2^ College of Mechatronics and Control Engineering Shenzhen University Shenzhen 518060 China

**Keywords:** cell handling, elasto‐inertial focusing, oscillartory microfluidics, soft actuators

## Abstract

Microfluidics can achieve the spatiotemporal manipulation of particles and cells in the microscale fluids, but highly relies on the accuracy of the pumping systems. To overcome this issue, a pump‐free, hydraulic‐amplification oscillatory microfluidic (PHOMF) device is presented, which can be actuated by fingers to handle particles and cells within the microchannel. The PHOMF device has a hydraulic‐amplification module for pressure transfer and a soft microchannel module for the generation of oscillatory flows. This is made possible by the periodic transfer of finger‐driven liquid pressure to the soft microchannel. This pressure causes the soft microchannel to deform and then drives the reciprocating flow of fluid volumes within the microchannel. In the oscillatory flow, particles and cells achieve single‐line focusing driven by the spatially accumulated inertial and elastic lift forces. The particle elasto‐inertial focusing theory in the PHOMF microchannel has been revealed. To demonstrate the system's practicality, the PHOMF device is utilized to achieve the early observation of platelet clots (3 min) and the rapid staining of cancer cells (8 min). The PHOMF device provides a miniaturized, inexpensive, and efficient detection tool for lab‐on‐a‐chip, and has the potential to become a mass‐produced, widely available, and convenient disease detection product.

## Graphical abstract

This study demonstrates a pump‐free, hydraulic‐amplification oscillatory microfluidic device, which generates oscillatory flows by deforming flexible microchannels through reciprocating finger pressing. The mechanism of elasto‐inertial focusing in the oscillatory flows is revealed, and early detection of drug‐treated platelet aggregates and rapid cell staining are enabled by this technique.

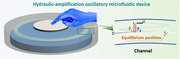

## 1 Introduction

Microfluidics precisely manipulates microparticles by the pump‐driven confined flow, which provides a robust and fault‐tolerant physical technique for the separation and enrichment of rare bioparticles. Due to the existence of scale constraints and inertial effects, the spontaneous spatial migration of microparticles realizes position‐differentiated focusing and ordering. The channel length required for inertial‐induced particle focusing is restricted by the flow velocity and the blockage ratio.^[^ ^1^ ^]^ Submicron‐ or nano‐particles (e.g., bacteria^[^ ^2^, ^3^ ^]^ and exosomes^[^ ^4^ ^]^) require a longer microchannel and a larger volumetric flow rate, which leads to excessive flow resistance and pressure within the microfluidic systems.

Complex microchannel structures (e.g., spiral,^[^ ^5^, ^6^ ^]^ serpentine,^[^ ^7^ ^]^ and contraction‐expansion^[^ ^8^, ^9^ ^]^) are designed to manipulate submicroparticles or nanoparticles by introducing secondary flow. The coupling of the additional lateral forces (i.e., secondary flow drag forces) with the inertial lift can accelerate the migration of submicroparticles or nanoparticles and shorten the physical channel length.^[^ ^7^, ^10^ ^]^ However, in the mentioned unidirectional flow, since the particle focusing is induced by the once‐only inertial lift and there is a non‐reusable space, it is restricted by microchannel geometries. By contrast, in oscillatory flow, the particle is reciprocally dragged and receives the inertial lift multiple times, enabling advantages in small particle manipulation and shorter channel lengths.^[^ ^11^, ^12^, ^13^ ^]^ The response of oscillatory flow relies on pump‐controlled systems that rapidly switch the flow direction at high frequencies, but these external components present non‐negligible challenges to the integration and miniaturization of the microfluidics equipment.

The strategy of integrating soft microfluidics and flexible actuators in soft robotics autonomously regulates fluid flow to avoid the use of pumping systems.^[^ ^14^ ^]^ Gas or liquid within the microfluidic network is pressurized by external energy (e.g., mechanical,^[^ ^15^ ^]^ electric,^[^ ^16^ ^]^ magnetic,^[^ ^17^ ^]^ and acoustic^[^ ^18^ ^]^), compelling it to squeeze the soft channels and actuators to enable the system to operate. Although these soft‐structured systems can drive the fluid flow in a unidirectional way, there is still an urgent need for a pump‐free approach to generate oscillatory flows so that the fluid can have an infinite flow in a length‐limited microchannel.

**Figure 1:**
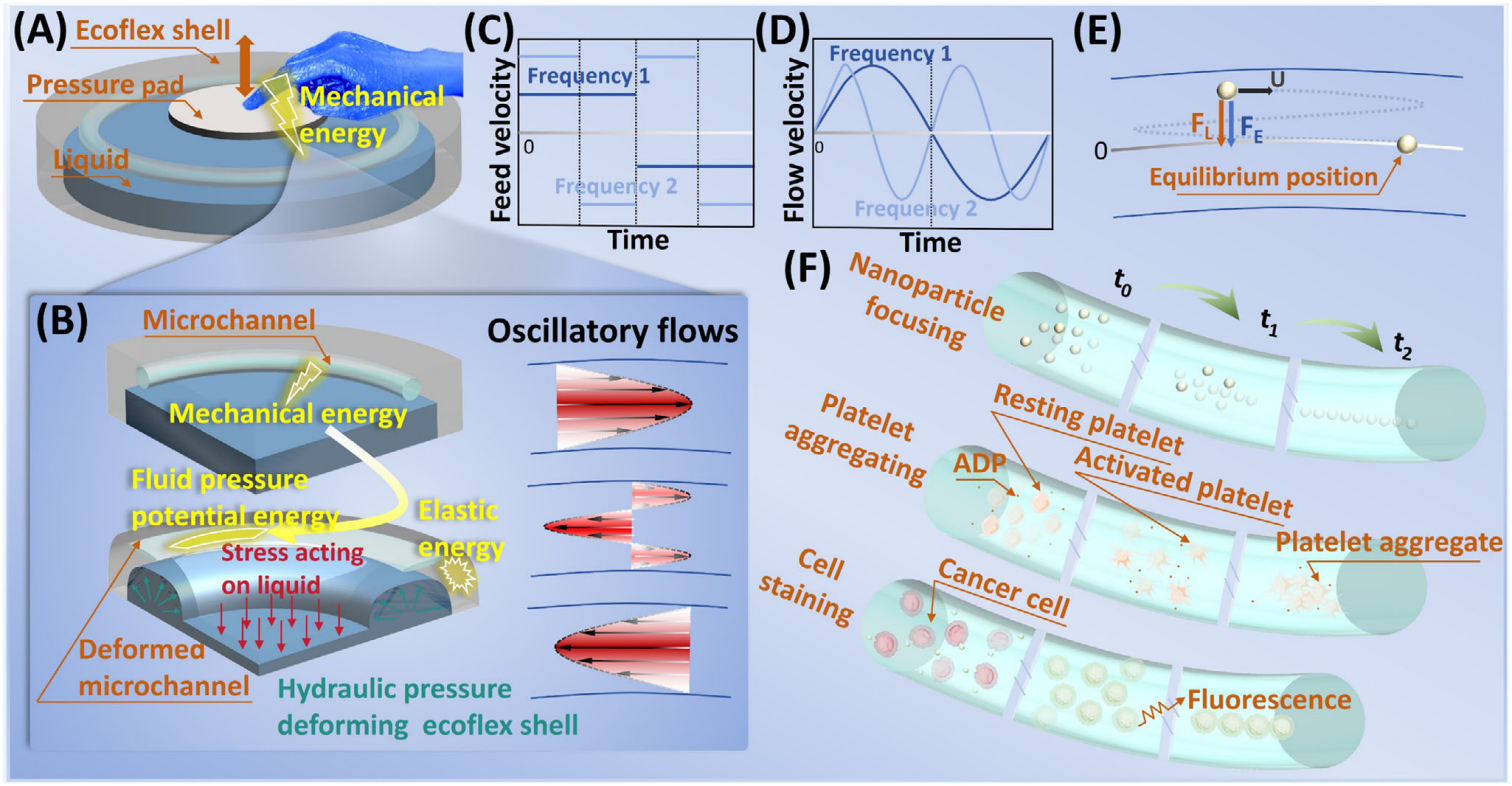
The working principle and applications of the PHOMF device. A) Schematic of the PHOMF device. The components of the PHOMF device include the Ecoflex elastic shell with a chamber, the working liquid in the chamber, the hollow microchannels embedded around in the Ecoflex shell, and the mechanical energy supply component. B) Partial schematic diagram. The transmission path of energy and the process of oscillatory flows. Microchannels are forced to deform within the liquid‐filled shell driven by mechanical energy, triggering the generation of oscillatory flow in the channel. C) The mechanical component of the feed motion provides energy to the PHOMF device. D) Response curve of the fluid flow velocity in the microchannel. E) Mechanism of particle migration. F) Applications of the PHOMF device include nanoparticle focusing, platelet aggregation, and cell staining.

Here, we report a pump‐free, hydraulic‐amplification oscillatory microfluidic (PHOMF) device for particle manipulation, providing power for the generation of oscillatory flow by hydraulic transmission and the deformation of soft microchannels (**Figure** **1**). To accomplish this, we use the thin membrane with microchannels as the chamber boundary, which deforms under the liquid pressure driven by fingers to drive the fluid flow. We apply periodic loads to the system to create a self‐sufficient and miniaturized oscillatory microfluidic platform. We delved into the working principle of the PHOMF device and the mechanism of particle focusing in oscillatory flow, and demonstrated proof‐of‐concept applications such as platelet dynamic coagulation and cancer cell staining.

## 2 Results and Discussion

### 2.1 Theory

Periodic frequency signals are input into the microfluidic system through external energy, which makes the fluid have a response of pressure gradient at different positions in the microchannel and triggers the generation of oscillatory flow. The pressure gradient drives the formation of the oscillatory flow field, and the velocity *v* in the main‐flow direction is solved by the simplified Navier–Stokes equations:

(1) $$\frac{\partial v}{\partial t}=Ae^{-i\omega t}+\nu \frac{\partial^{2}v}{\partial y^{2}}$$

where ω is the angular frequency, *A* is the amplitude of the pressure gradient, and ν is the fluid dynamic viscosity. The fluid flow in the channel is regarded as a no‐slip flow. The fluid velocity at the boundary is zero (*v* = 0 for *y* = ±*h*/2). The main‐flow velocity of the oscillatory flow is:

(2) $$v\left(y,t\right)=\frac{iA}{\omega }\;e^{-i\omega t}\left[1-\frac{\cos\left(ky\right)}{\cos\left(kh/2\right)}\right]$$

where $k=\sqrt{\frac{\omega }{v}}$. The average velocity of fluid in the channel cross‐section:

(3) $$U=\frac{1}{h}\int_{-h/2}^{h/2}v\left(y,t\right)dy=\frac{iA}{\omega }e^{-i\omega t}\left(1-\frac{2}{kh}\tan\left(\frac{kh}{2}\right)\right)$$

Particles are dragged by the oscillatory flow to move back and forth in a finite‐length microchannel. During this process, particles are repeatedly subject to inertial lift at the same position of the microchannel and migrate across the streamline to the equilibrium position. However, particles have multiple equilibrium positions in Newtonian‐fluid‐based inertial microfluidic systems, which is not applicable to applications in particle single‐line focusing (e.g., single‐cell analysis). For example, there is a 0.6‐times focusing band in the circular‐channel (i.e., Segre and Silberberg annulus),^[^ ^19^ ^]^ four equilibrium positions in the square‐channel,^[^ ^20^ ^]^ and two equilibrium positions in the rectangular‐channel.^[^ ^21^ ^]^ The concept of elasto‐inertial microfluidics is regarded as the gold standard for single‐stream 3D focusing in simple channel geometries.^[^ ^22^ ^]^ Particles are migrated to the channel geometric center in the non‐Newtonian fluid under the competition between the elastic and the inertial lift. The expressions of the inertial lift^[^ ^23^ ^]^ and the elastic lift^[^ ^24^ ^]^ are as follows:

(4) $$F_{L}=\frac{C_{L}\rho_{f}U^{2}a^{4}}{D^{2}}\;$$

(5) $$F_{E}=2C_{eL}a^{3}\eta_{P}\lambda \nabla {\dot{\gamma }}^{2}=8C_{eL}\eta_{P}\lambda U^{2}\frac{a^{3}}{D^{2}}$$

where *a* is the particle diameter, ρ_*f*_ is the fluid density, *U* is the average channel velocity, *D* is the diameter of the flow channel, η_*P*_ is the polymeric contribution to the solution viscosity, λ is the relaxation time of the fluid, and $\dot{\gamma }$ is the average fluid shear rate. *C_L_* and *C_eL_* are the inertial and the elastic lift coefficient, respectively.

For the particle elasto‐inertial focusing, the channel length required for particle focusing in the circular channel (unidirectional flow) is:^[^ ^25^ ^]^

(6) $$L_{unidirection}=\frac{\pi \log\left(\frac{D}{D-a}\right)D^{6}}{8a^{2}\lambda U}$$

The change of direction in oscillatory flows does not influence the directions of the inertial and elastic lift. The physical flow channel length for particle focusing under oscillatory flow has been deduced as (Supporting Information):

(7) $$L_{oscillatory}=\frac{3\pi \omega }{128}\;\frac{\mu }{C_{eL}\eta_{P}\lambda^{2}}\frac{1}{U^{3}}\frac{D^{9}}{a^{4}}\log\left(\frac{D}{D-a}\right)$$

The impact of the diffusion effect on particle migration needs to be reevaluated because of the low‐convection characteristic in viscoelastic fluids. The Péclet number is used to describe the relative importance of convection to diffusion in a particle‐fluid system:

(8) $$Pe=\frac{DU}{C_{diffusion}}$$

where *D* is the characteristic dimension of the microchannel, *U* is the characteristic fluid velocity. The diffusion coefficient is expressed using the Stokes–Einstein relation *C_diffusion_* = *k_B_* *T*/3πμ*a*, Among them, *k_B_* is Boltzmann's constant, which is ≈ 1.3806488 × 10^−23^ *J* · *K* ^−1^, and *T* is the absolute temperature. The diffusion of particles in viscoelastic fluids is expressed as:

(9) $$\begin{array}{ccc} Pe_{visc} & = & \frac{8C_{eL}\eta_{P}\lambda U^{2}}{k_{B}T}\cdot \frac{a^{3}}{D}=\frac{8C_{eL}\eta_{P}\lambda \mu^{2}}{k_{B}T\rho^{2}}\cdot \beta^{-1}\cdot Re_{P}^{2}\\ & = & C\cdot C_{eL}\cdot \beta^{-1}\cdot Re_{P}^{2} \end{array}$$

where particle blockage ratio β = *a*/*D*, particle Reynolds number *Re_P_* = ρ*Ua* ^2^ · (μ*D*)^−1^, and *C* is a constant when the fluid properties and the system medium remain unchanged. The particle suspension with 1000 ppm Polyethylene oxide (PEO, λ is 6.8 ms) works at room temperature, and *C* can be estimated to be 303.95 × 10^5^ *kg* · *m* ^2^.

### 2.2 Working Principle

For the PHOMF device (Figure 1A,B), the oscillatory flows are generated for particle focusing through the periodic deformation of the soft microchannel actuated by hydraulic pressure. This is achieved by squeezing the liquid in the sealed chamber, and the liquid forces the thin membrane with microchannels to deform. This leads to the reciprocating flow of the pre‐injected particle suspension in the microchannel and forms an oscillatory flow. Particles driven by the oscillatory flow migrate toward the center of the microchannel under the continuous action of the elasto‐inertial lift, so as to achieve the submicron‐ or nano‐particles manipulation in a relatively short microchannel.

Microchannels are fabricated by soft‐lithographic techniques (Figure S1, Supporting Information). Copper wires (30–200 µm) as molds are buried in uncured Ecoflex and then removed after the Ecoflex curing to form microchannels. The flexible membrane with microchannel and the Ecoflex supporting substrate are assembled to form a sealed chamber through edge encapsulation. The working fluid is filled into the chamber to act as the hydraulic transmission medium of the PHOMF device. When the flexible membrane is in a relaxed state, the chamber can hold 4 mL of water. Finally, the particle suspension is injected into the microchannel before the microfluidic system's operation.

The excitation source of external feeding motion transmits mechanical energy to the working fluid to make it displaced (Figure 1C). The working fluid forces the flexible membrane to undergo a fully reversible elastic deformation and continuously store and release elastic potential energy. The microchannels arranged circumferentially in the flexible membrane are subjected to isotropic pressure and thus squeeze the fluid in the microchannel (Figure 1B). The periodic external energy stimulation on the microfluidic system will inevitably cause the fluid to flow in a reciprocating harmonic manner because of the continuity of energy transfer (Figure 1D).^[^ ^26^ ^]^ On the premise of not considering energy loss, the external mechanical energy is completely transformed into the pressure potential energy of fluid flow. We assume that a sinusoidally varying force (*F*(*t*)  = *A_E_* *sin*(ω_*E*_ *t*)) does work on the PHOMF system. At any moment (t_0_), the energy of the microfluidic system, the elastic energy of the flexible membrane, and the fluid pressure potential energy are:

(10) $$E=\int_{0}^{t_{0}}F\left(t\right)dt=\frac{A_{E}}{\omega_{E}}\left[1-\cos\left(\omega_{E}t_{0}\right)\right]$$

In the direction‐changing harmonic Stokes flows (i.e., oscillatory flow), the particles migrate to the equilibrium position where the net lift (inertial and elastic lift) is zero under elastic and viscous shear (Figure 1E). The elasto‐inertial oscillatory flows have a relatively low Reynolds number, which provides the possibility of confining the particles within the microscopic perspective in a wide frequency range. Therefore, it is convenient to observe the dynamic processes of the bioparticles development in physiological or pathological samples, which is difficult to achieve with steady flows. In this paper, we apply oscillatory flows of PHOMF to the nanoparticles focusing and the dynamic monitoring of platelet aggregation and cell staining (Figure 1F).

### 2.3 Characterization of the PHOMF Device

**Figure 2:**
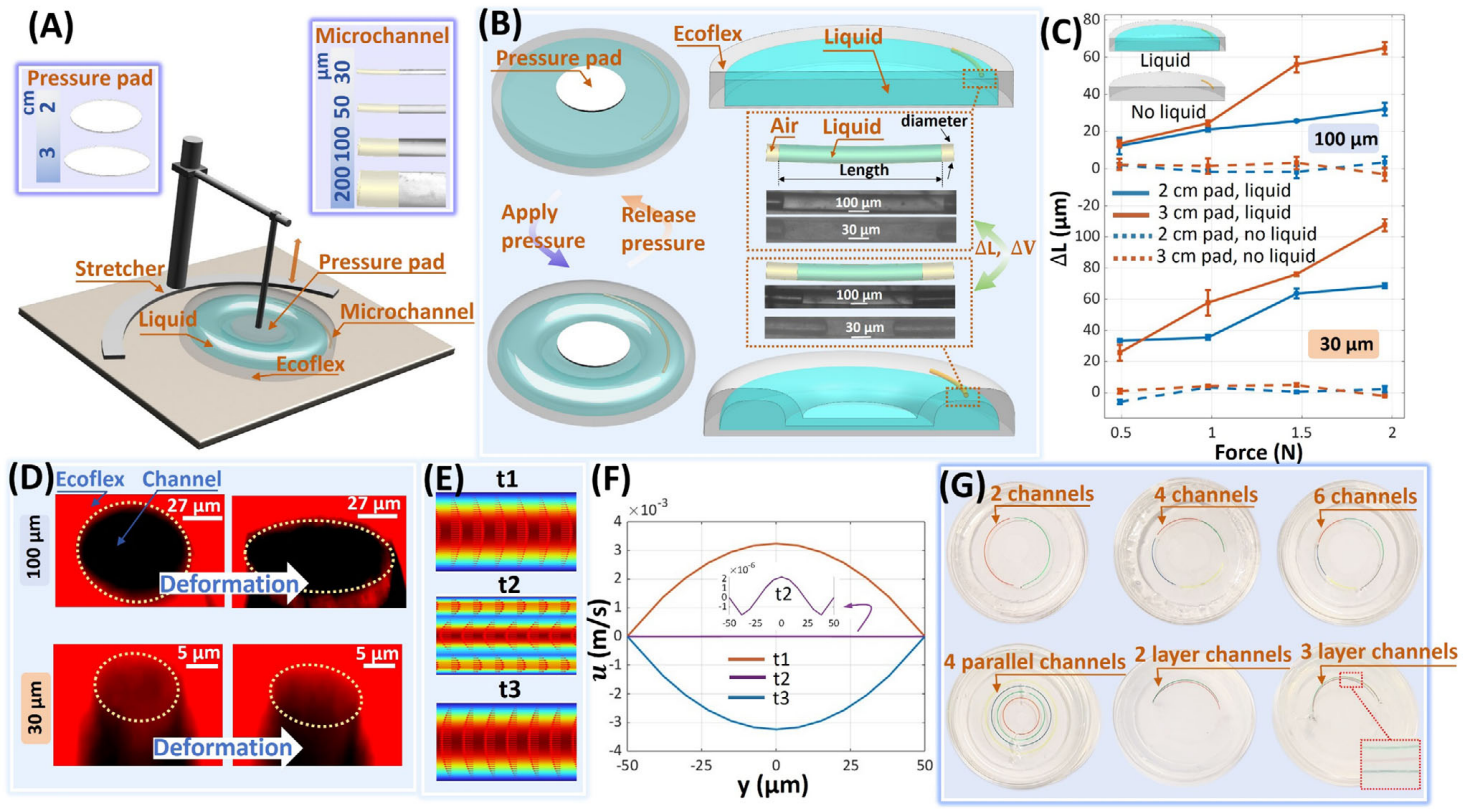
Structural characteristics of the PHOMF device. A) The various components of the system. The external auxiliary power supply elements include programmable joysticks and a rigid pressing pad. The working elements of the microfluidic system include the working fluid and a flexible membrane with a microchannel. B) The morphology of the microchannel under the application and release of pressure. C) The length changes of 30 and 100 µm channels under different loads are captured and measured by gas‐liquid refractive index imaging under different working conditions (working fluid, channel diameter). D) The cross‐sectional changes of the 30 and 100 µm channels were photographed by confocal microscopy imaging. E) Numerical simulation of oscillatory flow in the PHOMF device (30 µm channel). Forward flow (t1), turning (t2), and reverse flow (t3). F) Statistical chart of velocity distribution in the microchannel cross‐section. G) Arrange the microchannels in multiple forms.

The changes in the 3D topography of the microchannel during the deformation of the flexible membrane, which are related to the dynamic process of micro‐volume fluid flow, are used to analyze the oscillatory flow in the PHOMF device. We configured the PHOMF device with 30, 50, 100, and 200 µm channels and used the circular press plates with a diameter of 2 and 3 cm (**Figure** **2**A). We measured the impact of the application of external loads on the microchannels from two dimensions to analyze fluid flow. 1) The change in the microchannel length was obtained through side‐view microscopic imaging with refractive resolution at the gas‐liquid interface. 2) The change in the microchannel cross‐section was acquired by confocal microscopy imaging.

In the PHOMF device, the isotropic microchannel under pressure from a rigid plate cannot be deformed when there is no working fluid in the chamber (i.e., no hydraulic pressure). In contrast, when hydraulic pressure is present, an increase in load can cause greater deformation of the microchannel (Figure 2B,C). This is because mechanical stress generates pressure and displacement on the liquid medium, which can cause the microchannel in the Ecoflex shell to deform. When the flexible membrane is under pressure, the size of the rigid pressing pad determines 1) the distance between the recessed edge of the membrane and the microchannel, 2) the volume of the working fluid that is squeezed into the non‐pressed area. We arranged the microchannel at a distance of 4 cm from the center of the system. Compared with the 2 cm pressing pad, the 3 cm pressing pad drives more working fluid away from the system center and is closer to the microchannel, resulting in greater deformation of the microchannel. In addition, the experimental results show that under the same load, the cross‐sectional change of the 100 µm channel is more significant than that of the 30 µm channel, while the deformation of the channel length is the opposite (Figure 2D). The reason is that the larger hollow microtube or microchannel inside the membrane is subjected to greater radial forces. When subjected to external forces, the 100 µm channel undergoes a transformation into a super‐elliptical shape (major axis: minor axis = 2.1). In contrast, for the 30 µm channel, the ratio of the major axis to the minor axis is merely 1.5. The variation of the channel length plays a crucial role in the generation of oscillatory flow. Under the same working conditions, the 30 µm channel has more significant oscillatory flow than the 100 µm channel and can provide a longer physical channel length for particle focusing.

We conducted numerical simulations on the oscillatory flow in the 30 µm channel within the PHOMF device (Figure 2E,F). Fluid experienced forward flow (t1), turning(t2) and reverse flow (t3). In this process, the fluid flow velocity ($∼2\times 10^{-6}\;m/s$) during turning is much lower than that of the Poiseuille flow ($∼3\times 10^{-3}\;m/s$). Therefore, we neglected the effect of the fluid on the particles at the turning moment in the oscillatory flow.

Oscillatory microfluidics can effectively reduce the required flow channel length for focusing, but its obvious drawback is that the throughput of the system for processing particle suspensions decreases. Parallelization is a common and extremely effective method to increase the throughput of microfluidic devices.^[^ ^27^, ^28^ ^]^ The PHOMF device has ample space and provides a method for the diversified parallel arrangement of microchannels. The planar‐layout annular channels (3, 4, and 6 channels), concentric parallel channels, and multi‐layer channels in 3D layout (2 or 3 layers) have been fabricated, demonstrating the flexibility of this PHOMF system (Figure 2G).

### 2.4 Single‐Line Focusing of Particles and Cells in the PHOMF Device

**Figure 3:**
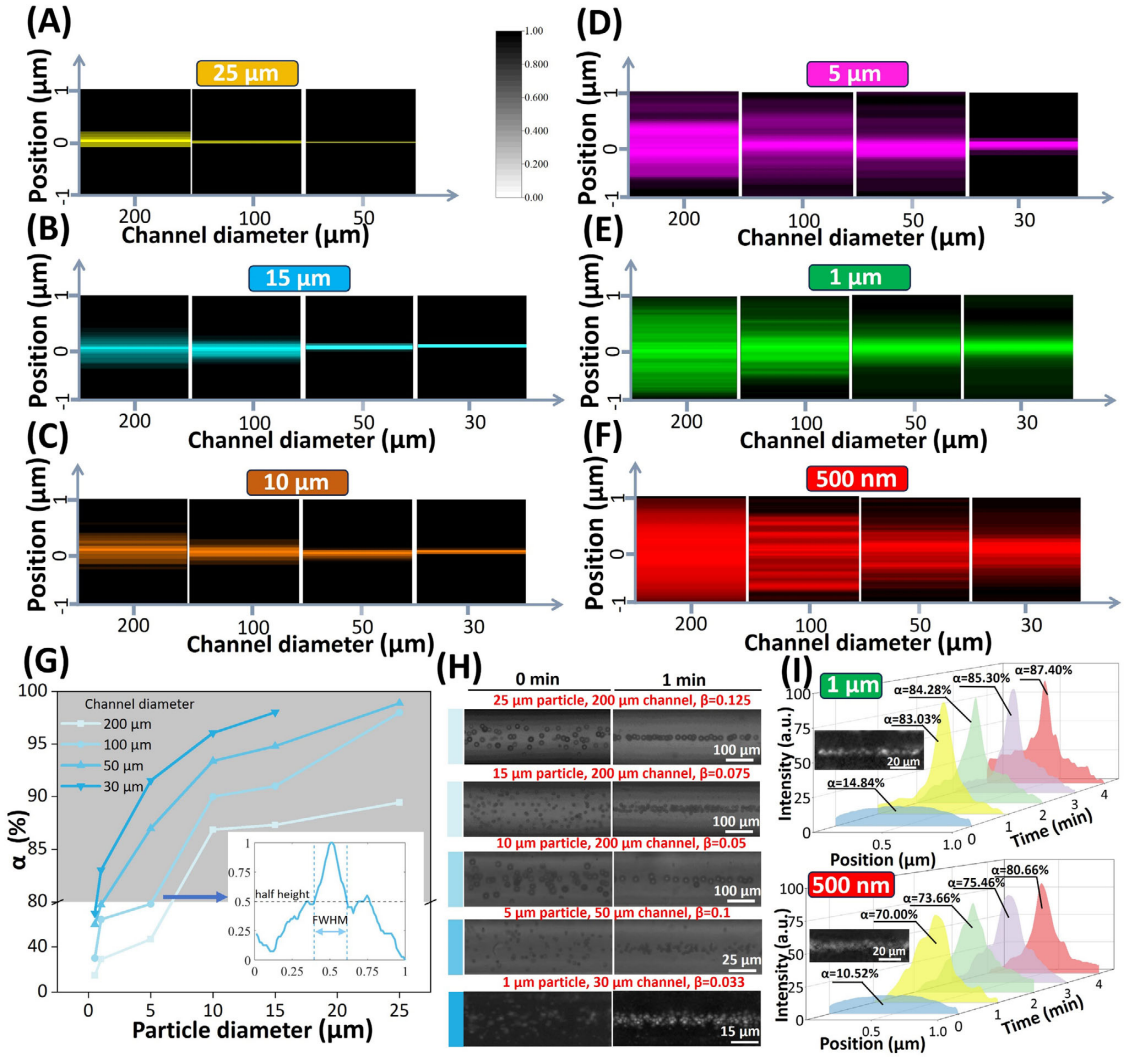
Particle focusing in the PHOMF device. The PHOMF device operated at a frequency of 3 Hz for 1 min with a PEO concentration of 1 Kppm. A–F) Color‐coded images showing the positional distributions of 25, 15, 10, 5, 1 µm, and 500 nm particles in 200, 100, 50, and 30 µm microchannels after 1 min operation of the PHOMF device at 3 Hz frequency. The particle positions have been normalized by the microchannel diameter. Darker colors indicate higher particle density at corresponding positions. G) The particle focusing efficiency in the microchannels was quantified using α = (1‐normalized FWHM) × 100%. The inset illustrates the normalized FWHM of 5 µm particles in a 100 µm microchannel after 1 min of PHOMF operation. The region with α > 80% (gray‐shaded area) was defined as the effective particle focusing zone. H) Time‐lapse microscopic images of particle distributions before (0 min) and after (1 min) PHOMF device activation. Dark‐field microscopy was employed for 1 µm particles, while bright‐field imaging was used for all other particle sizes. I) With increasing operation time of the PHOMF device, the focusing behavior of 1 µm and 500 nm particles in the 30 µm channel, including particle distribution and focusing efficiency (α). The dark‐field images reveal the focusing state of 1 µm and 500 nm particles after 4 min of operation in the PHOMF device.

After characterizing the PHOMF device, we aim to achieve pump‐free particle manipulation through finger‐driven motion. The geometric dimensions of the microchannel and the particle size are key factors for effectively manipulating particles. We fabricated PHOMF devices with microchannels of 200, 100, 50, and 30 µm. Suspensions of particles with sizes of 25, 15, 10, 5, 1 µm, and 500 nm were loaded into 5 mL syringes and introduced into the microchannels. The PEO concentration in the particle suspension was 1 Kppm. Here, particles in the 5–25 µm range were non‐fluorescent, whereas the 1 µm and 500 nm particles exhibited fluorescence. The motion trajectories of the particles were observed and captured using an inverted microscope equipped with a high‐speed CCD camera. Time‐lapse images were stacked using ImageJ. After operating the PHOMF device at a frequency of 3 Hz for 1 min, the position distribution of particles in the microchannels was plotted as a color‐coded image, as shown in **Figure** **3**A–F. The detailed procedure for generating the color‐coded image is provided in Figure S2 (Supporting Information).

For microchannels with smaller diameters, the particles formed tighter focus streams (Figure 3A–F). Additionally, larger particles demonstrated better focusing performance in the channels. We characterized the particle distribution using normalized full width at half maximum (FWHM) and quantified the focusing efficiency as α = (1‐normalized FWHM) × 100%. The normalized FWHM of 5 µm particles in the 100 µm microchannel is shown in Figure 3G, with α = 78.35% in this case. We defined α = 80% as indicating complete particle focusing in the microchannel (Gray area). 10, 15, and 25 µm particles achieved focusing with α > 80% in all four channel types (Figure 3G,H). This successful focusing occurred because the blockage ratio β ≥ 0.05, enabling the particles to experience sufficient inertial and elastic lift forces within 1 min. This demonstrates that the oscillatory flow generated by the PHOMF device overcomes the conventional inertial microfluidics limitation, where particle focusing typically requires β ≥ 0.07.^[^ ^29^ ^]^ We further evaluated the focusing performance of the PHOMF device using three distinct cell types representing different size ranges: PC12 cells (Adrenal tumor cells, 8–10 µm), SW620 cells (Colorectal cancer cells, 10–20 µm), and MDA‐MB‐231 cells (Breast cancer cells, 15–26 µm). The positional distribution of these cells was analyzed in a 100 µm channel before and after 1 min of device operation, as shown in Figure S3 (Supporting Information). Experiments used a 2000 ppm PEO solution at 5 Hz operating frequency. Under oscillatory flow driving, all these cells were efficiently focused within the microchannel.

To elucidate the particle migration mechanism, we utilized direct numerical simulation to calculate the elasto‐inertial lift coefficient, which revealed that 10 µm particles converge to the 100 µm channel center at a steeper slope than 5 µm particles (Figure S4, Supporting Information). Furthermore, we employed a Lagrangian particle tracking method to establish a force model matching experimental parameters, simulating the trajectories of 5 and 10 µm particles along both the flow direction (*x*‐axis) and transverse direction (*y*‐axis), as shown in Figure S5 (Supporting Information). The results demonstrate that five initially uniformly distributed particles reciprocate along the x‐axis under the influence of direction‐changing viscous drag force (F_drag_ ∝ a^2^), while along the y‐axis they migrate toward the microchannel center via lift forces, achieving sequential focusing from outer to inner positions. Using this method to manipulate particles reduces the required microchannel for 10 µm particles by 2.35 × 10^3^ times compared to unidirectional flow, and for 5 µm particles by 2.48 × 10^2^ times. Moreover, the channel physical length for focusing particles in this report (2.09 mm) has reached a new low bound compared to other oscillatory microfluidic particle manipulation techniques.^[^ ^11^, ^12^ ^]^ Table S1 (Supporting Information) summarizes the comparative performance of our PHOMF device versus other microfluidic technologies in terms of required channel length for particle focusing and operating flow rates/Reynolds numbers, highlighting the advantages of the PHOMF platform. This liberates particle manipulation from the constraints of flow channel geometries, facilitating the simplification of microfluidic chip design processes.

However, 1 µm particles achieved effective focusing (α > 80%) only in 30 µm channels (Figure 3G,H), while 500 nm particles exhibited broad particle streams in 30–200 µm channels (Figure 3F). Based on the previously established physical intuition of particle migration, the obstruction of diffusive motion to the elasto‐inertial migration is more pronounced for nanoparticles compared to microparticles. We explored the behavior of 1 µm (β = 0.033) and 500 nm particles (β = 0.017) in 30 µm channels by prolonging the PHOMF device's runtime. The theoretical focusing time for 500 nm particles is 1.98 times that of 1 µm particles (Equation 6). However, in reality, with the obstruction of Brownian motion to particle focusing, this multiple is greater than 4 (Figure 3I). The 500 nm particles barely reached 80.66% focusing efficiency after 4 min of operation, compared to 1 µm particles, which attained 83.03% efficiency in just 1 min. During the operation of the PHOMF device, the particle Reynolds number (*Re_p_*) is 1.10 × 10^−4^ for 1 µm particles and 2.04 × 10^−6^ for 500 nm particles. The average value of 0.5 of the lift coefficient *C_eL_* is used to estimate the Peclet number of the particles. The Péclet numbers are $Pe_{1\;\mu m}∼O\left(10^{0}\right)$ for 1 µm particles and $Pe_{500\;nm}∼O\left(10^{-3}\right)$ for 500 nm particles. At this extremely low *Re_p_*, the diffusion of nanoparticles is quite significant compared to convection (*Pe* ≪ 1). This means that it takes a longer time to achieve the focusing of nanoparticles in the PHOMF device. We optimized the focusing of 500 nm particles in 30 µm microchannels by adjusting the PEO concentration in the particle suspensions, the operating frequency of the PHOMF device, and the microchannel layout in the PHOMF device, as shown in the following.

### 2.5 Optimization of Nanoparticle Focusing

**Figure 4:**
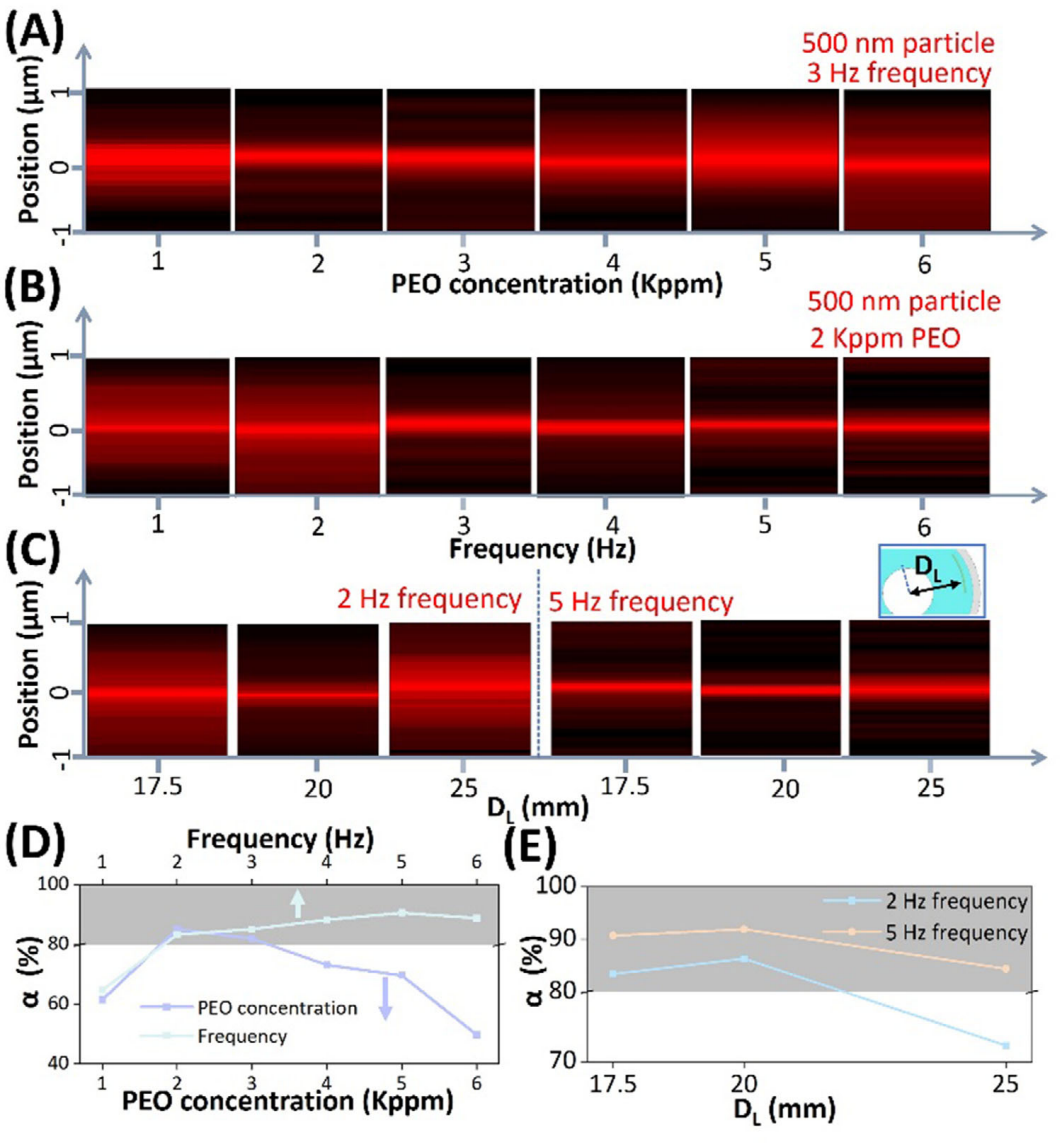
Optimization of 500 nm particles focusing in PHOMF devices. The operational duration of PHOMF devices is 1 min. A) The positional distribution of 500 nm particles after migration in suspensions with varying PEO concentrations (1, 2, 3, 4, 5, 6 Kppm) at an operating frequency of 3 Hz. B) The positional distribution of 500 nm particles in 2 Kppm PEO suspension under different device operating frequencies. C) Impact of microchannel layout in PHOMF devices on the positional distribution of 500 nm particles in 2 Kppm PEO suspension at operating frequencies of 2 and 5 Hz. Here, the microchannel layout specifically refers to the distance between the flow channel centerline and the PHOMF device's geometric center (D_L_). D) Particle focusing efficiency (α) under varying PEO concentrations and operating frequencies. E) Particle focusing efficiency (α) across different microchannel layouts.

In this section, we optimize the focusing efficiency of 500 nm particles in a 30 µm microchannel by adjusting the operating conditions, including PEO concentration, device operating frequency, and microchannel layout. We prepared particle suspensions with PEO concentrations of 1, 2, 3, 4, 5, and 6 Kppm. After operating the PHOMF device at a frequency of 3 Hz for 1 min, the positional distribution of particles in the microchannel is shown in **Figure** **4**A. The 500 nm particles demonstrated significantly enhanced elasto‐inertial focusing in 2 and 3 Kppm PEO solutions compared to the 1 Kppm PEO solution. This phenomenon is attributed to the stronger contribution of F_E_ with greater elasticity number (EI) in the 2 and 3 Kppm PEO solutions, while the influence of F_L_ diminished due to the lower Re compared to the 1 Kppm PEO solution. However, as the PEO concentration increased, the particle stream transitioned from a dispersed state to a tightly focused one, and then back to a dispersed state. This occurs because higher PEO concentrations result in lower Re, which reduces the axial flow distance of particle suspensions in oscillatory flow, consequently driving particle migration away from the channel centerline.^[^ ^30^ ^]^ The optimal particle focusing effect was achieved at a PEO concentration of 2 Kppm, with α reaching 85.26% (Figure 4D).

Furthermore, we adjusted the operating frequency of the PHOMF device to further enhance the focusing effect of 500 nm particles in the 2 Kppm PEO solutions, testing frequencies of 1, 2, 3, 4, 5, and 6 Hz (Figure 4B). As the working frequency increases, the particle stream gradually becomes more compact. At higher operating frequencies, the fluid oscillates more rapidly, causing it to traverse a longer physical flow path within 1 min. Under the increased frequency of the fluid, particles experience stronger axial drag forces, leading to more intense motion. Meanwhile, in the lateral direction, particles are subjected to prolonged elasto‐inertial lift forces, resulting in improved focusing performance within the microchannel. At a frequency of 5–6 Hz, α remains stable at around 90% (Figure 4D).

We investigated the effect of channel layout on particle focusing using a 2 Kppm PEO solution at operating frequencies of 2 and 5 Hz. We fabricated PHOMF devices with microchannel centerlines located at varying distances (D_L_ = 17.5, 20, and 25 mm) from the geometric center of the device. The color‐coded images of particle distribution in Figure 4C demonstrate that both excessively close and distant channel positions relative to the device center are detrimental to particle focusing. This occurs because the elastic membrane is constrained by compression plates and fixed by the dish, resulting in significantly greater deformation of the microchannel positioned at the membrane's central region during PHOMF device operation. At an operating frequency of 5 Hz, the particle focusing efficiency (α) reaches 92% in the PHOMF device with D_L_ = 20 mm (Figure 4E).

### 2.6 Biological Applications of PHOMF Devices

**Figure 5:**
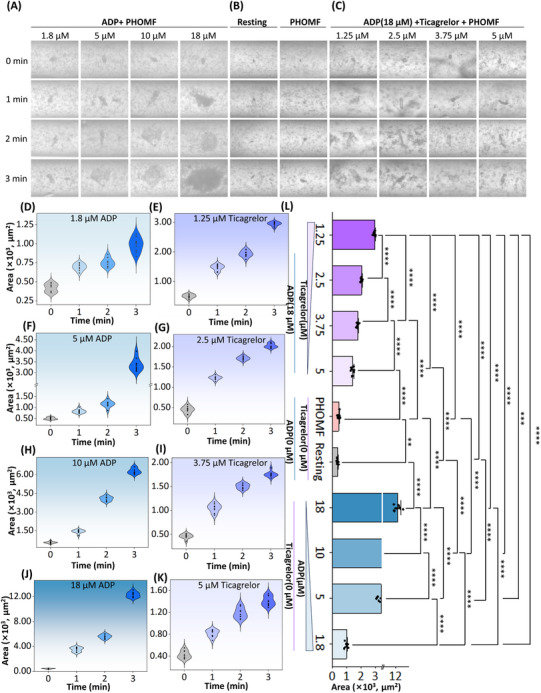
Platelet aggregation assays were conducted using the PHOMF platform. The PHOMF device was operated for 3 min at a frequency of 5 Hz, with a PEO concentration of 2 Kppm, and the microchannel layout on the device had a D_L_ of 20 mm. Time‐dependent platelet aggregation responses under various pharmacological treatments were characterized, including: A) ADP‐induced activation (agonist stimulation), B) baseline controls comprising both static incubation (resting) and oscillatory shear flow conditions without pharmacological intervention, and C) ticagrelor‐mediated suppression (antiplatelet therapy). The concentration of introduced ADP was kept constant at 18 µm. D, F, H, J) Platelet aggregation profiles under varying ADP concentrations (1.8, 5, 10, and 18 µm) in the PHOMF device. E, G, I, K) Platelet aggregation profiles under different ticagrelor concentrations (1.25, 2.5, 3.75, 5 µm) in the PHOMF device. L) Statistical analysis (*t*‐test) of platelet area following 3 min. ^*^ *p* < 0.05, ^**^ *p* < 0.01, ^***^ *p* < 0.001, ^****^ *p* < 0.0001.

In order to verify the diversity of the PHOMF device in the application of bioparticles, we applied this device to the dynamic monitoring of platelet aggregation and cell staining. Coagulation tests related to atherosclerotic thrombosis are a fundamental requirement for disease diagnosis.^[^ ^31^, ^32^ ^]^ However, their long turnaround time (> 4 h) and excessive sample consumption (≈3 mL) pose a huge challenge for point‐of‐care testing and clinical diagnosis with small sample sizes.^[^ ^33^ ^]^ To validate the feasibility of antiplatelet aggregation testing on the PHOMF platform, we designed a comprehensive comparative study with three experimental conditions (**Figure** **5**): 1) procoagulant treatment, 2) anticoagulant treatment, and 3) negative controls (including both PHOMF‐processed and resting samples without drug treatment). Time‐lapse microscopy was employed to quantitatively monitor temporal variations in platelet aggregation area. The operating frequency of the PHOMF device is 5 Hz, with a channel size of 200 µm and a PEO concentration of 2 Kppm.

For the procoagulant treatment group, platelets incubated with 1.8, 5, 10, and 18 µm adenosine diphosphate (ADP) were aggregated under oscillatory flow (Figure 5A). ADP, as a common natural agonist, will also be spontaneously released during the process of platelet activation and transmit signals to adjacent platelets.^[^ ^34^, ^35^, ^36^ ^]^ Through the interaction of platelet membranes, platelets approach each other and form stable aggregates. The oscillatory flow increases the contact possibility of blood clots during this process, which enables the blood to coagulate more quickly and become more viscous. Under oscillatory flow conditions in the PHOMF device, platelet aggregation occurred significantly faster than in static slide cultures without pharmacological stimulation (Figure 5B,L). A dose‐dependent potentiation of platelet aggregation was observed with increasing ADP concentrations, demonstrating the agonist's concentration‐responsive effect (Figure 5D,F,H,J). Following a 3‐min exposure to 18 µm ADP in the PHOMF platform, platelet aggregates achieved an area of 1 2330 ± 370 µm^2^. This demonstrated a significant difference (*p* < 0.0001) compared to control measurements (in PHOMF device) of 445 ± 102 µm^2^ (Figure 5L). Meanwhile, platelets are focused at the center of the microchannel under the hemodynamic shear of elasto‐inertial oscillatory flow, presenting an excellent perspective for dynamic detection.

In the anticoagulant treatment group, platelets pre‐incubated with varying concentrations of ticagrelor (1.25, 2.5, 3.75, and 5 µm) were individually introduced into the microchannels. As a fast‐acting oral antiplatelet agent demonstrating enhanced therapeutic efficacy, ticagrelor has been widely adopted in clinical practice following comprehensive development trials.^[^ ^37^, ^38^ ^]^ The G‐protein‐coupled receptor P2Y_12_, activated by ADP, serves as the central mediator of platelet aggregation, thrombus amplification, and stabilization through intracellular signaling cascades.^[^ ^37^ ^]^ Therapeutically, ticagrelor exerts its antiplatelet effects via selective P2Y_12_ receptor antagonism, effectively suppressing platelet activation and aggregation to mitigate thrombotic event risks. Time‐lapse microscopic images demonstrating platelet morphological evolution under varying ticagrelor concentrations are presented in Figure 5C and Figure S6 (Supporting Information). Ticagrelor demonstrated dose‐dependent suppression of platelet aggregation, with progressive inhibition observed across escalating concentrations (*p* < 0.0001 for 1.25 µm vs 3.75 µm) (Figure 5E,G,I,K). Oscillatory shear flow significantly enhanced platelet exposure to ticagrelor. Compared to static controls, platelets pre‐incubated with 5 µm ticagrelor exhibited markedly suppressed aggregation, with platelet‐covered area increasing only to 1411.88 ± 108.35 µm^2^ after 3 min of dynamic treatment (*p* < 0.0001 vs untreated group) (Figure 5L). We trained two male and two female operators to perform platelet coagulation assays, effectively eliminating operator‐dependent factors in PHOMF device operation through standardized protocols (Figure S7, Supporting Information). A comparative evaluation of coagulation times in platelet coagulation assays between the PHOMF platform and other existing analytical systems is summarized in Table S2 (Supporting Information), highlighting PHOMF's enhanced capacity to resolve dynamic clotting processes under physiologically relevant shear conditions. The time needed for thrombus growth in experiments using the PHOMF device (3 min) is significantly shorter than the 20 min duration required by electrode‐driven microfluidics^[^ ^33^ ^]^ and the 12–15 min needed by unidirectional laminar microfluidics.^[^ ^39^ ^]^ Moreover, the PHOMF demonstrated excellent operational stability and the ability to operate for an extended period by the long‐term performance test (Figure S8, Supporting Information). We firmly believe that the PHOMF device will serve as an instant blood coagulation detection method and is expected to provide a microfluidic technology that is rapid, requires a small sample volume, and is convenient for dynamic observation for the diagnosis of thrombotic diseases (e.g., myocardial infarction, stroke, deep vein thrombosis).

Cell staining enables the identification and characterization of cellular structures, bringing possibilities for clinical diagnostics.^[^ ^40^ ^]^ Here, we demonstrated the process of cell straining in our PHOMF device. The fluorescent dye (DiI) is mixed with the cancer cell solution and then injected into a 100 µm channel. Under the mixing effect and shearing effect of the oscillatory flow, the fluorescent dye colors the cell membrane. The complete mixing of fluorescent dye and DI water within 4 min verified the mixing effect of the oscillatory flow (Figure S9, Supporting Information). This leads to a higher cell staining efficiency in the microchannel of the PHOMF device than in the static state (Figure S10, Supporting Information). After the 8‐min test, the fluorescence signal intensity of cancer cells under the action of the oscillating flow is ≈1.8 times that under the static condition. Table S2 (Supporting Information) provides a comparative summary of our PHOMF platform versus other cell staining technologies, demonstrating that the PHOMF device achieves shorter staining time (8 min) and requires less fluorescent dye (1 µL). The combination of this rapid cell staining technique and electroporation transfection technology^[^ ^41^ ^]^ is expected to accelerate the phagocytosis of drugs and provide convenience for disease diagnosis and drug research, and development.

## 3 Summary and Outlook

In this work, we have developed a pump‐free oscillatory microfluidic device that generates oscillatory flow by the periodic deformation of the microchannel actuated by fingers. The finger‐driven mechanical energy is turned into the elastic potential energy of the flexible membrane through hydraulic transmission. This elastic potential energy is then converted into fluid pressure potential energy within the microchannels, triggering a tiny amount of fluid volume flow. The periodic change of energy makes the fluid flow direction alternate back and forth, thus generating an oscillatory flow. After demonstrating the working principle of the PHOMF device, our experiments on particle elasto‐inertial self‐focusing and theoretical analyses show that under extremely low particle Reynolds number $Re_{p}∼O\left(10^{-4}\right)$, within 1 min, small particles with a blockage ratio β  =  0.033 can achieve single‐line focusing. However, for an even lower particle Reynolds number *Re_p_* < *O*(10^−6^), the diffusion effect of particles is significantly increased $Pe∼O(10^{-3})$, meaning that nanoparticles require a longer time to achieve focusing. We investigated the distribution of nanoparticles in microchannels by adjusting parameters such as microchannel diameter, PEO concentration, operating frequency, and channel layout to optimize particle focusing. Optimal particle focusing (α ≈ 92%) was achieved at a channel diameter of 30 µm, PEO concentration of 2 Kppm, operating frequency of 5 Hz, and a channel position 80 mm from the PHOMF device center. As a proof of concept, we demonstrated effective focusing of three distinct cell lines in the microchannel: PC12 cells, SW620 cells, and MDA‐MB‐231 cells. Platelet coagulation assays using agonists and anticoagulants under PHOMF‐induced oscillatory flow revealed the platform's key advantages: small sample (1.48 nL) and short turnaround time (3 min). Furthermore, under the shearing and mixing effects of the oscillatory flow, the fluorescence signal of cancer cells in the PHOMF device was 1.8 times higher than the static staining (8‐min staining).

The PHOMF device overcomes the limitations of the traditional pump‐controlled systems and provides the microfluidics community with a new portable and easy‐to‐manufacture platform, which is expected to shorten the time cycle of point‐of‐care testing. We believe that the PHOMF device will be further applied to process complex samples containing submicron/nanoscale bioparticles, such as bacteria, exosomes, and DNA macromolecules. However, the PHOMF device currently suffers from low throughput in sample processing. By integrating parallelized microchannel architectures, the system's throughput could be multiplicatively enhanced. Although our PHOMF device eliminates the need for external pumping systems, it relies on manual operation. By implementing a liquid metal‐based battery‐powered pumping mechanism, continuous liquid delivery for up to 12 h can be achieved.^[^ ^42^ ^]^ In future work, we will focus on upgrading the PHOMF platform to realize a fully self‐driven, pump‐free integrated microfluidic system.

## 4 Ethics Statement

This study was approved by the Institutional Animal Care and Use Committee of Shenzhen University Medical School (IACUC‐202400039).

## Conflict of Interest

The authors declare no conflict of interest.

## Supporting information



Supporting Information

## Data Availability

The data that support the findings of this study are available from the corresponding author upon reasonable request.
