# Supplementary material for: A Pump‐Free, Hydraulic‐Amplification Oscillatory Microfluidic Device for Continuous Particle and Cell Manipulation
Source: Adv Sci (Weinh). 2025 May 23;12(30):e07041. doi: 10.1002/advs.202507041 (PMC12376699; doi:10.1002/advs.202507041)
Supplement: Supplementary file 1 — Supporting Information [file ADVS-12-e07041-s001.docx]

**Supporting information**

**for**

A pump-free, hydraulic-amplification oscillatory microfluidic device for continuous particle and cell manipulation

Yong Liu^1,2†^, Mingyi Liang^1†^, Shanshan Xu^1^, Sheng Yan^1,2*^

^1^ Institute for Advanced Study, Shenzhen University, Shenzhen, China

^2^ College of Mechatronics and Control Engineering, Shenzhen University, Shenzhen, China

^†^ Y.L. and Y.M.L. contributed equally to this work.

^*^ Corresponding authors: shengyan@szu.edu.cn

**This PDF file includes:**

The length of the microchannel required for particle focusing.

Preparation of the elasto-inertial medium.

Preparation of particle.

Cell culture and preparation of cell suspensions.

Preparation of platelet samples.

Preparation of solutions for cell staining.

Experimental Setup.

Design and fabrication of a hydraulic-amplification oscillatory microfluidic device (PHOMF).

Plotting a colour-coded image.

Cell focusing in the PHOMF device.

Calculation of lift and motion trajectory.

Time-lapse micrographs of platelet coagulation and corresponding quantitative statistical plots.

Operator-dependent factors affecting PHOMF device performance.

Long-term performance test.

Mixing effect.

Cell staining.

Comparison of PHOMF devices with other technologies in particle/cell focusing, Platelet aggregation, and cellular staining.

References.

**The length of the microchannel required for particle focusing.**

By balancing the elastic lift force and the Stokes drag, the particle migration velocity in the flow direction ($U_{L}$) within a microchannel can be calculated:

$$F_{stokes}=3\pi\mu aU_{L} (1)$$

$$F_{E}=8C_{eL}\eta_{P}\lambda U^{2}\frac{a^{3}}{D^{2}} (2)$$

$$U_{L}=\frac{8C_{eL}\eta_{P}\lambda U^{2}a^{2}}{3\pi\mu D^{2}} (3)$$

The circular-channel length required for particle focusing within a non-Newtonian fluid (unidirectional flow)^[1]^:

$$L_{unidirection}=\frac{\pi\log\left( \frac{D}{D-a} \right)D^{6}}{8a^{2}\lambda U} (4)$$

The channel length (physical channel length) required for particle focusing in an oscillatory flow with angular frequency $\omega$:

$$L_{oscillatory}=\frac{2t}{T}\cdot L_{unidirection}=\frac{\omega}{\pi}\cdot\frac{D}{2U_{L}}\cdot\frac{\pi\log\left( \frac{D}{D-a} \right)D^{6}}{8a^{2}\lambda U}=\frac{3\pi\omega}{128}\frac{\mu}{C_{eL}\eta_{P}\lambda^{2}}\frac{1}{U^{3}}\frac{D^{9}}{a^{4}}\log\left( \frac{D}{D-a} \right) (5)$$

where，$T$ is the period of the oscillatory flow.

**Preparation of the elasto-inertial medium.**

Poly (ethylene oxide) (PEO, 2MDa; Macklin) was dissolved in deionized (DI) water to prepare a concentration of 6000 ppm, which has excellent biocompatibility. Subsequently, the 6000 ppm PEO suspension was mixed with particle/cell samples at volume ratios of 5:1, 2:1, 1:1, 1:2, and 1:5 to achieve final PEO concentrations of 5000 ppm, 4000 ppm, 3000 ppm, 2000 ppm, and 1000 ppm, respectively. The effective relaxation time of the 1000 ppm PEO solution is 6.8 ms, and its zero-shear viscosity is 2.3 mPa·s.^[2]^

**Preparation of particle.**

Six sets of spherical polystyrene microbeads (25 μm, 15 μm, 10 μm, 5 μm, 1 μm, and 500 nm) with varying diameters were used to evaluate the effects of particle size/blockage ratio on elasto-inertial focusing in the PHOMF device. The 25 μm, 15 μm, 10 μm, 5 μm particles are non-fluorescent particles, and the 1 μm and 500 nm particles are fluorescent particles (PS-Nano, Zhichuan). 25 μm, 15 μm, 10 μm, 5 μm, and 1 μm particles were separately dispersed in the prepared 1000 ppm PEO elasto-inertial medium. 500 nm particles were dispersed in PEO elasto-inertial media with concentrations of 6000 ppm, 5000 ppm, 4000 ppm, 3000 ppm, 2000 ppm, and 1000 ppm, respectively, to investigate the effects of viscoelastic fluid properties on particle focusing. The prepared particle suspensions had a particle-to-mass ratio of 0.08-0.2% (w/w). Tween 20 (Sigma-Aldrich, Cat. #P9416) was added as a surfactant at 0.1% (w/w) to prevent particle aggregation.

**Cell culture and preparation of cell suspensions.**

The PC12 cells (Adrenal tumor cells, 8-10 μm), SW620 cells (Colorectal cancer cells, 10-20 μm), and MDA-MB-231 cells (Breast cancer cells, 15-26 μm) purchased from Procell Life Science & Technology Co., Ltd. (Wuhan, China) were cultured in 10 cm LABSELECT dishes with DMEM medium containing 10% heat-inactivated fetal bovine serum (VIVA CELL) and 1% penicillin-streptomycin solution (VIVA CELL) at 37°C under 5% CO₂, harvested at ~90% confluence via microscopic observation, washed with 1 mL PBS (VIVA CELL) to remove debris, detached using 1 mL trypsin for 2 min in the incubator, neutralized with 2 mL complete medium, centrifuged at 1000 rpm for 3 min at room temperature in 15 mL tubes, and finally resuspended in 1 mL fresh complete DMEM medium for downstream use. The cultured cell suspension was mixed with 6000 ppm PEO solution at a 1:2 volume ratio to prepare a 2000 ppm PEO-based cell solution. The prepared 2000 ppm PEO-cell solution was preloaded into the PHOMF device to validate it focusing performance.

**Preparation of platelet samples.**

To maximize coagulation detection efficiency, platelets and platelet aggregates were enriched from rat whole blood via density gradient centrifugation.^3^ Firstly, healthy whole blood was collected using 3.2% citrate as an anticoagulant. The concentration of citrate, which is a commonly used anticoagulant, can significantly affect the clinical results of prothrombin time (PT) and activated partial thromboplastin time (aPTT) tests. Previous studies have demonstrated that a citrate concentration of 3.2% is recommended for use in clinical coagulation tests.^4^ Following blood collection, platelet-rich plasma separation was achieved through differential centrifugation (3,000 rpm, 3 min, room temperature) using a clinical-grade mini centrifuge (Yooning, Mini-10K+, Hangzhou). Then, we incubated the platelet-rich plasma with the agonist /anticoagulant. Specifically, platelet-rich plasma was incubated with ADP (agonist, C10H13N5Na2O10P2, BioboMei, MW:417.16) at concentrations of 1.8 μM, 5 μM, 10 μM, and 18 μM, respectively, at a volume ratio of 200 μL of blood to 20 μL of ADP solution for 1 minute. Moreover, 18 μM ADP was mixed with ticagrelor (anticoagulant, C23H28F2N6O4S, Solarbio, Lot. No.2503240001) at concentrations of 1.25 μM, 2.5 μM, 3.75 μM, and 5 μM, respectively, at a volume ratio of 10 μL of ADP solution to 20 μL of ticagrelor solution. Subsequently, the plasma was mixed with the resulting solution at a volume ratio of 200 μL of plasma to 20 μL of the mixture and incubated for 1 minute. The drug-treated platelets were then supplemented with phosphate-buffered saline (PBS). Subsequently, we mixed the solution with 6000 ppm PEO at a 2:1 volume ratio and stirred the mixture using a mixer (JOANLAB, VM-300, Shenzhen) to prepare a blood solution based on 2000 ppm PEO for subsequent platelet coagulation assays.

**Preparation of solutions for cell staining.**

Membrane staining reagents (DiI, C1991S, Beyotime, orange-red under fluorescence microscopy with a maximum excitation wavelength of 549 nm and a maximum emission wavelength of 565 nm) was mixed with breast cancer cells (MDA-MB-231), then combined with 6000 ppm PEO solution at a 2:1 volume ratio to prepare a 2000 ppm PEO-based cell-dye working solution. DiI is a carbocyanine dye with long hydrophobic hydrocarbon chains. After incorporation into the cell membrane, it undergoes lateral diffusion, gradually staining the entire cell membrane. Fluorescent signals from cells were observed using an inverted fluorescence microscope (Ti2-U, Nikon) during PHOMF device operation. The prepared solution was loaded onto a glass slide and observed under an inverted fluorescence microscope for control experiments.

**Experimental Setup.**

The high-speed inverted microscope (Ti2-U, Nikon) was used to record the particles or bioparticles trajectories in microchannel at a frame rate of 3000 fps. A confocal microscope (A1 HD25, Shanghai Nikon) was used to observe the cross-sectional changes of the microchannels in the PHOMF device under working conditions. At 20× objective magnification, the fluorescence microscope was controlled by the Z-stack mode of the Zeiss ZEN software to take multiple sets of fluorescence micrographs along the z-axis direction with a scanning step of 0.3 µm from top to bottom. X-axis and y-axis positions remained unchanged. Each image was taken with a pixel resolution of 1024×1024. 3D images were reconstructed using ZEN software. The commercial software COMSOL Multiphysics 6.0 was used to analyze the transient flow field in the PHOMF device and simulate the particles trajectories. Image J (National Institutes of Health) was used for post-processing and image analysis.

**Design and fabrication of a hydraulic-amplification oscillatory microfluidic device (PHOMF).**

The design concept of the PHOMF device is to generate oscillatory flow by causing deformation of the microchannel through hydraulic pressure. The PHOMF device consists of an Ecoflex sheet with microchannel (top layer), an Ecoflex fundus (bottom layer), and working fluid (water). The microchannels in the PHOMF device are fabricated by the soft-lithographic techniques. (Fig. S-1). The 30 μm, 50 μm, 100 μm and 200 μm copper wires were buried in the Ecoflex mixture (Ecoflex 00-31, Smooth-On) and then pulled out to form microchannels, and this part was used as the top layer of the PHOMF device. In addition, the cured Ecoflex mixture was left without any other treatment and this part was used as the bottom layer of the PHOMF device. The two parts were edge-bonded with Ecoflex to form a cavity. Holes were punched at the edge of the device and the working liquid was injected into the chamber. The manufacturing process is executed in detail as described below.

**Top layer.** (1) In order to create a chamber for accommodating the working fluid, a circular acrylic plate with a thickness of 2 mm (chamber mold) is pasted in the middle of the dish. Uncured Ecoflex 00-31 covers the bottom of the dish and submerges the surface of the mold. (2) Ecoflex spreads and solidifies after 4 hours to form an “Ecoflex sheet”. (3) The copper wire is straightened and wound into a circle, then placed at the center of the mold. (4) Then, a layer of Ecoflex is poured to bury the copper wire. (5) The copper wire is pulled out after the Ecoflex cures. (6) The Ecoflex sheet is taken out of the dish. (7) Flip this Ecoflex sheet over so that the groove faces upward. This part serves as the top layer of the PHOMF device, preparing for the subsequent assembly.

**Bottom layer.** Pour Ecoflex (1) and let it cure for 4 hours (2) to form an Ecoflex fundus. Use this part as the bottom layer of the PHOMF device.

**Assembly.** Apply Ecoflex to the edges of the top layer (8) and bond it with the bottom layer (9). (10) After the Ecoflex cures, these two parts are firmly bonded together. (10) The working fluid is injected into the chamber.


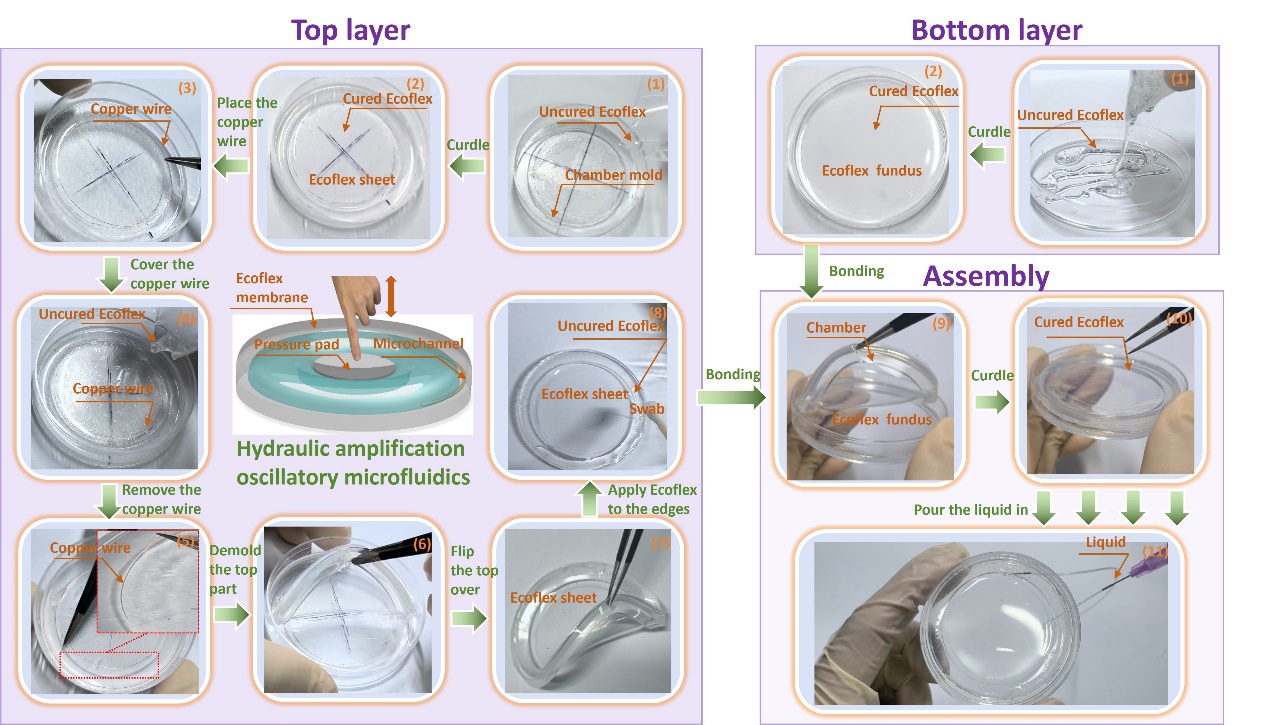
Fig. S-1 The fabrication of the PHOMF device is divided into three parts: the Ecoflex top layer with embedded microchannels (featuring grooves), the Ecoflex fundus, and the working fluid in the cavity.

**Plotting a colour-coded image.**

To better visualize particle focusing performance in the PHOMF microchannel, we generated a color-coded image quantifying the lateral (cross-channel) positional distribution of particles. The detailed plotting procedure is illustrated in Fig. S-2. First, raw images were acquired via microscopy, collecting 100 transverse grayscale distribution profiles (grayscale value versus lateral position) at 100 distinct axial locations along the microchannel (Fig. S-2, A and B). Next, the averaged distribution profile was calculated from these 100 individual profiles, followed by normalization of both lateral channel positions and grayscale values to generate the composite curve (Fig. S-2, C). This curve was then inverted, with higher values now representing increased particle density at corresponding lateral positions in the channel (Fig. S-2, D). Thus, we obtained a false-color image representing the position distribution of particles within the microchannel (Fig. S-2, E).


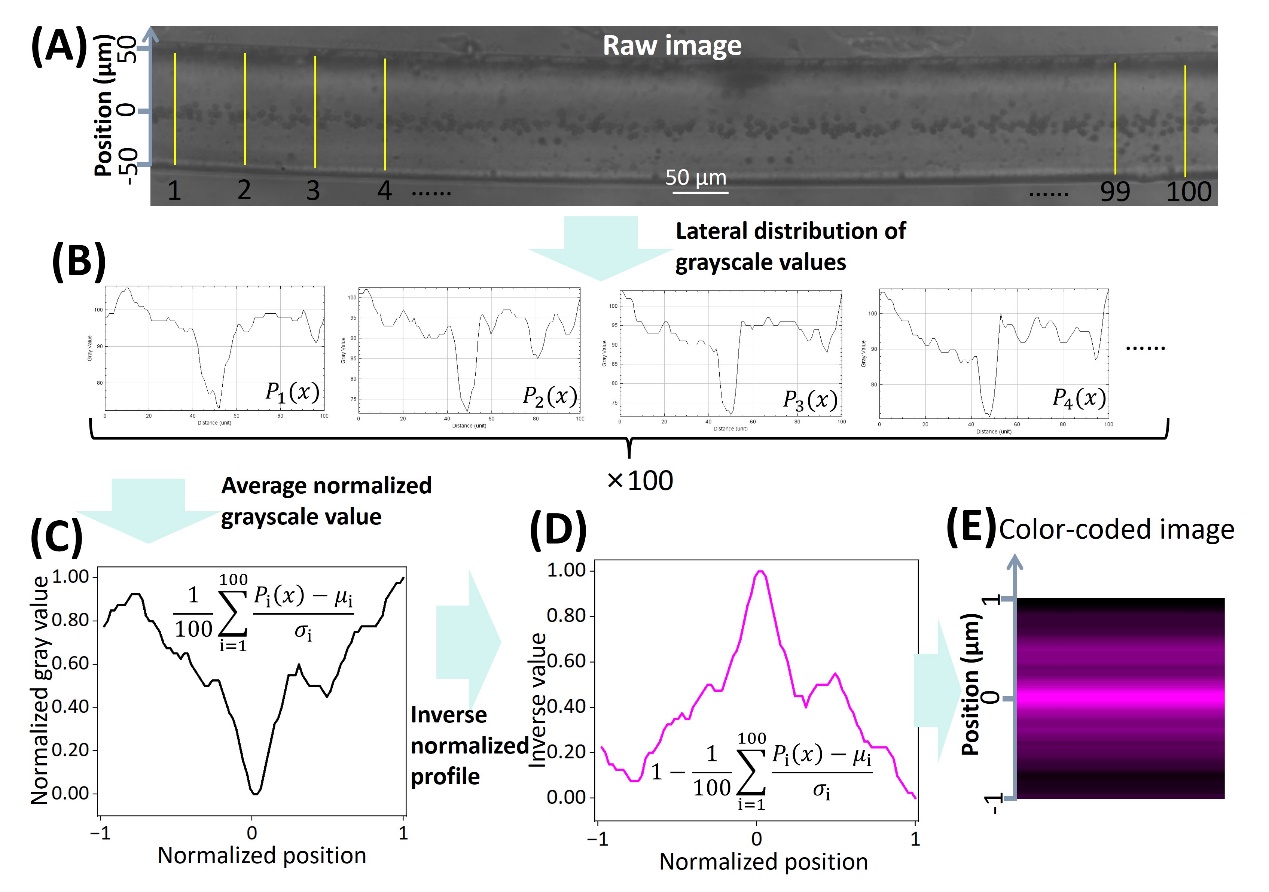


Fig. S-2 The procedure for generating a colour-coded image. (A) The raw image is captured using an inverted microscope, showing 5 μm particles suspended in a 1 Kppm PEO solution flowing through a 100 μm channel of a PHOMF device operating at 3 Hz frequency, after 1 minute of operation. (B) The grayscale distribution curve of 100 positions in the microchannel. (C) The average distribution curve of 100 grayscale distribution curves. (D) The inversion curve used to reflect the focusing effect of particles. (E) Visualization of the processed data as a colour-coded image.

**Cell focusing in the PHOMF device.**

Cultured cells were suspended in PBS with 2000 ppm PEO, introduced into the PHOMF device, and their position distribution within the microchannel was observed using an inverted microscope. The study employed three cell lines: PC12 cells (Adrenal tumor cells, 8-10 μm), SW620 cells (Colorectal cancer cells, 10-20 μm), and MDA-MB-231 cells (Breast cancer cells, 15-26 μm). We performed experiments using 100 μm microchannel, with the PHOMF device operating at 5 Hz for 1 minute. The position distribution of cells in the microchannel before (0 min) and after (1 min) PHOMF operation is shown in Fig. S-3. After 1 min of PHOMF operation, all three cell types were effectively focused toward the central region of the microchannel. These results demonstrate the robust capability of the PHOMF device to manipulate cells across varying sizes.


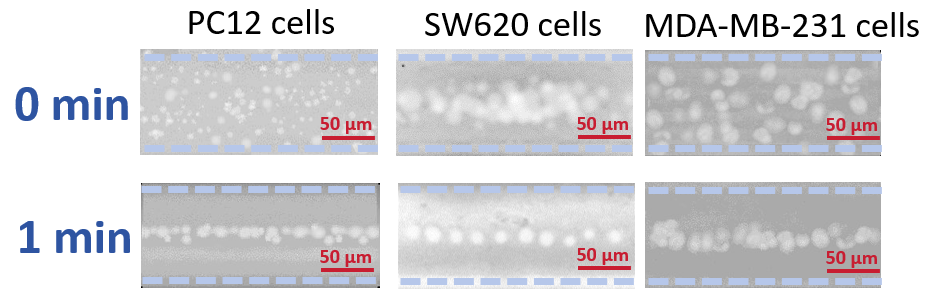


Fig. S-3 The PHOMF device achieved focusing of three distinct cell types: PC12 cells (Adrenal tumor cells, 8-10 μm), SW620 cells (Colorectal cancer cells, 10-20 μm), and MDA-MB-231 cells (Breast cancer cells, 15-26 μm). The PHOMF device was operated under the following parameters: 2000 ppm PEO solution, 5 Hz actuation frequency, and 100 μm microchannel diameter. The positional distribution of cells in the microchannel of the PHOMF device before startup (at 0 min) and 1 minute after operation.

**Calculation of lift and motion trajectory.**

The two-dimensional inertial lift field is calculated using the fluid mechanics module in COMSOL. The inertial lift force ($F_{L}$) and the channel dimensions (R) are normalized:

$$r^{*}=\frac{2r}{R} (6)$$

$$C_{L}=F_{L}\cdot\left( \frac{R^{2}}{4\rho U^{2}a^{4}} \right) (7)$$

where, $r$ is the radial position of the particle in the circular channel, and $R$ is the radius of the channel. For particles of 5 μm and 10 μm, the net lift force is zero at the center of the channel, causing the particles to converge at the channel center and achieve single-line focusing (Fig. S-4). The flow conditions and microchannel geometry in the simulations were kept identical to the experimental setup. We mapped the inertial lift field onto the microchannel cross-section and computed particle trajectories using the Lagrangian particle tracking method. The trajectories of 5 μm and 10 μm particles in both axial (x) and radial (y) directions within the microchannel are illustrated in Fig. S-5. Particles initially released from five distinct radial positions exhibited oscillatory motion along the axial direction while gradually focusing toward the channel centerline in the radial direction under inertial lift forces. Compared to 5 μm particles, the 10 μm particles migrated faster toward the channel centerline under stronger inertial lift forces.


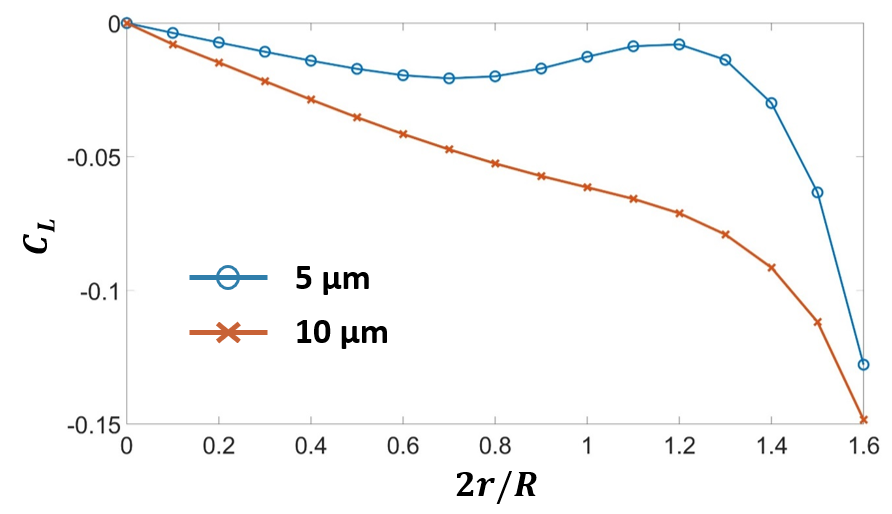


Fig. S-4 The radial lift distribution of the particle in the channel (100 μm) is calculated through direct numerical simulation.


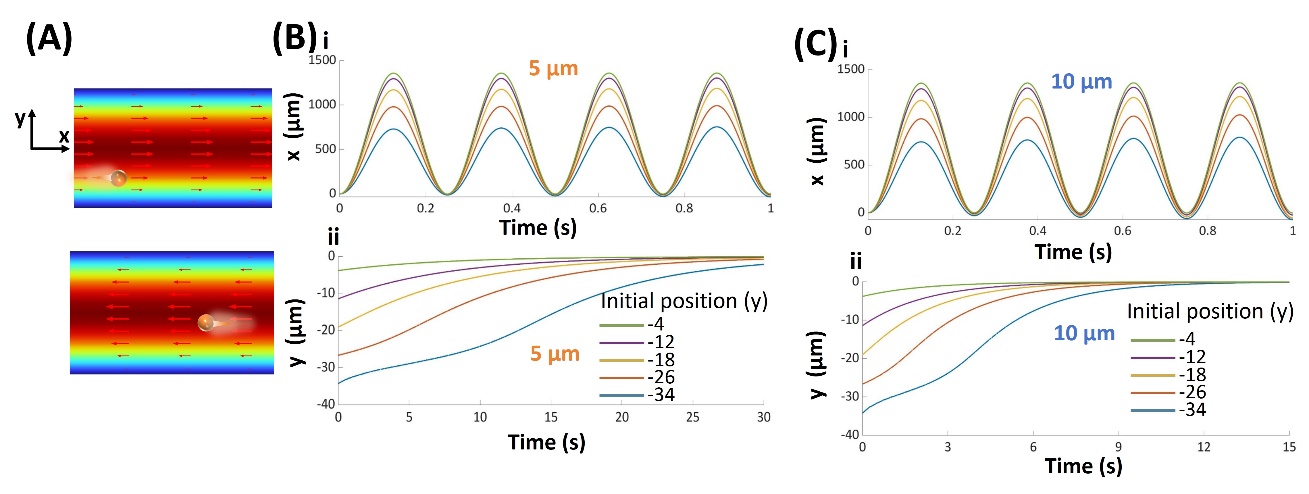


Fig. S-5 (A) Schematic diagram of particle motion in oscillatory flow. Simulation of the motion of 5 μm (B) and 10 μm (C) particles in the flow direction (x-axis) and lateral direction (y-axis).

**Time-lapse micrographs of platelet coagulation and corresponding quantitative statistical plots.**

We observed the platelet coagulation process in blood samples using the PHOMF device, and performed size measurements and statistical analysis on multiple platelet aggregates to quantify coagulation dynamics (Fig. S-6A). Blood samples premixed with 18 μM ADP and varying concentrations of ticagrelor were infused into the PHOMF microchannel, with the device operated at 5 Hz in 2 Kppm PEO solution for 3 minutes. Platelet aggregation was increasingly inhibited with higher concentrations of ticagrelor (Fig. S-6B).


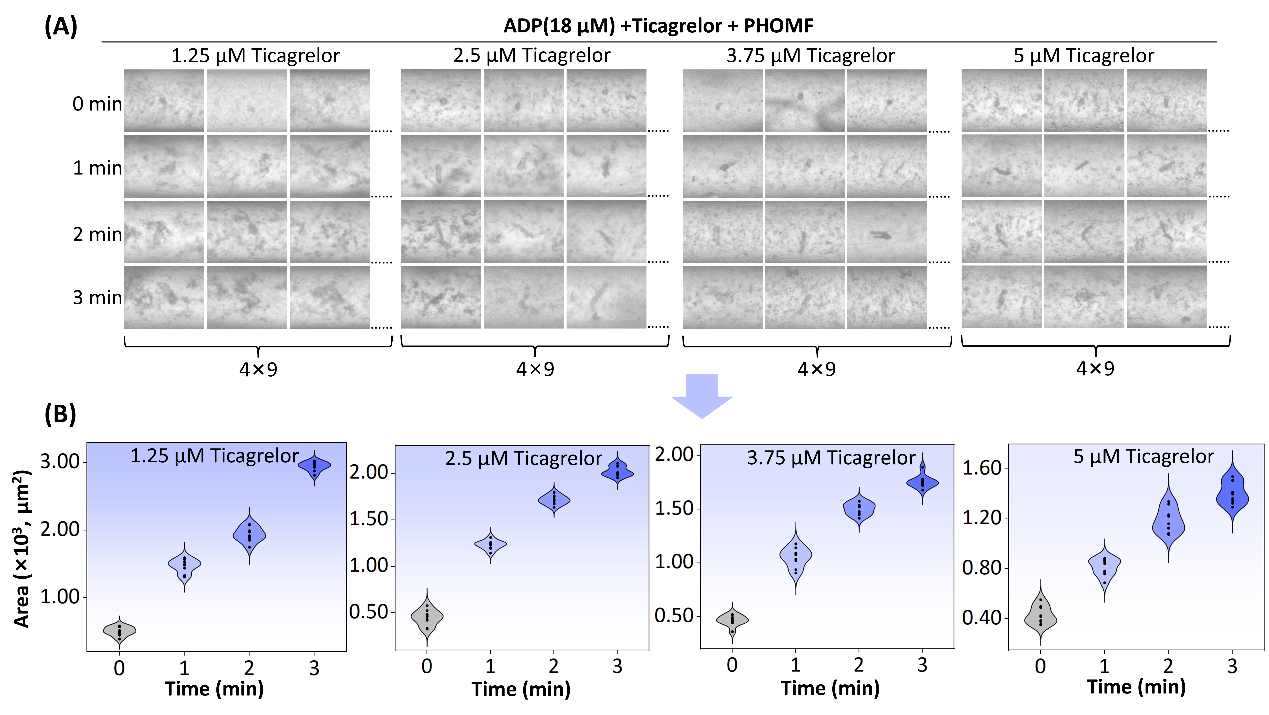


Fig. S-6 Under varying anticoagulant concentrations (1.25, 2.5, 3.75, 5 μM) combined with PHOMF device operation, platelet aggregate sizes at different time points were measured using ImageJ and presented as violin plots for statistical analysis. Whole blood pretreated with 18 μM ADP and varying concentrations of ticagrelor was incubated for 1 minute before being transferred into the microchannel of the PHOMF device. (A) Bright-field microscopic images of platelets were acquired at 0, 1, 2, and 3 minutes of PHOMF operation using an inverted microscope. (B) For each time point, data from 9 individual platelets were collected and presented as violin plots to illustrate distribution characteristics.

**Operator-dependent factors affecting PHOMF device performance.**

To investigate the influence of individual finger-pressing variability in the hydraulically amplified oscillatory microfluidic device proposed in this study, we recruited a cohort of four adult volunteers (two adult males and two adult females) to assess platelet aggregation dynamics under standardized operating parameters. Under standardized protocols, four volunteers performed finger-actuated operation of the hydraulically amplified oscillatory microfluidic device at a controlled frequency of 5 Hz, with real-time guidance from our research team. During the experimental procedure, minor deviations from the target 5 Hz frequency occurred owing to natural variations in manual finger actuation among different operators. The experimental parameters were set as follows: PEO concentration at 2000 ppm, channel diameter at 200 μm, and total operation time limited to within 3 minutes. Each operator repeated the experiment 10 times. We performed coagulation assays in the hydraulically amplified oscillatory microfluidic device using two distinct platelet samples: (1) platelets incubated with 1.8 μM ADP, and (2) platelets co-incubated with 18 μM ADP and 5 μM ticagrelor.

Real-time monitoring of aggregate morphology was performed via high-speed microscopy, with quantitative size measurements recorded at precisely controlled time intervals (0 min, 1 min, 2 min, and 3 min post-activation). The experimental results revealed no statistically significant differences in platelet aggregate size across the four tested individuals. Independent t-test analysis demonstrated that inter-individual variability had negligible impact on coagulation outcomes (*p*>0.05) (Fig S-7). The experimental results conclusively demonstrate that the hydraulically amplified microfluidic platform eliminates operator-induced variability in device performance when operated under standardized protocols, ensuring reproducible outcomes across diverse user groups. Despite slight variations in operators' finger-pressing strokes, frequency, and pulses, the experimental results demonstrated remarkable consistency, indicating the PHOMF device's excellent operational robustness.


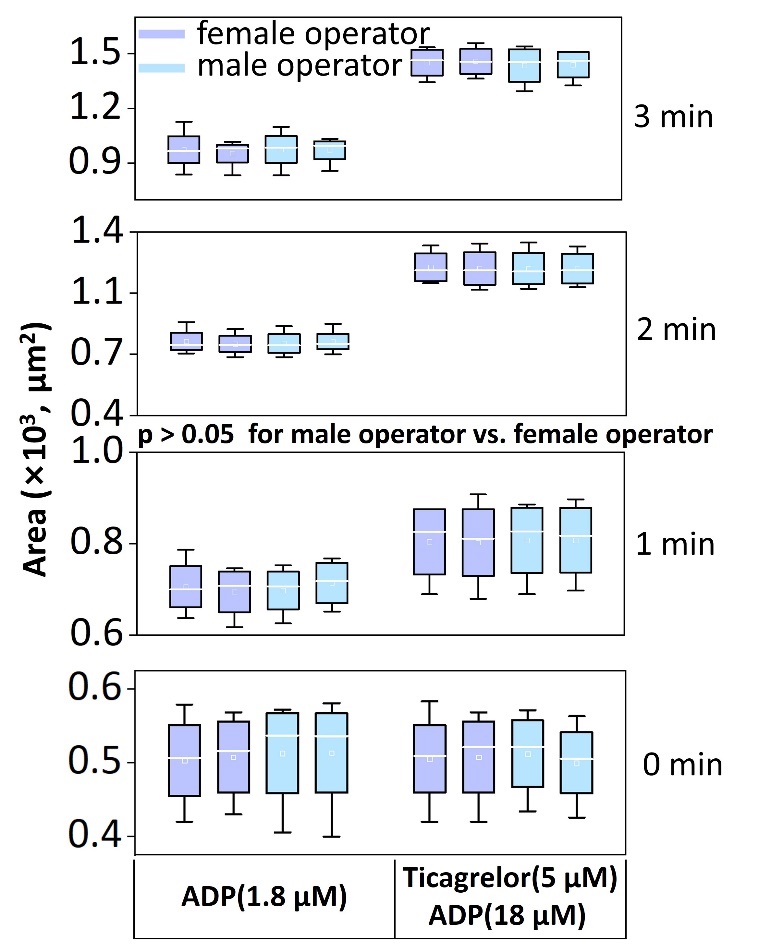


Fig. S-7 Platelet coagulation assays were performed by four trained operators (two male and two female) using the PHOMF device according to standardized protocols. The study comprised two treatment groups: (1) ADP-only (1.8 μM) and (2) combination of ticagrelor (5 μM) with ADP (1.8 μM) pre-incubated platelet samples. Each operator repeated the experiment 10 times. Platelet aggregate areas were acquired via microscopic imaging at 0 min, 1 min, 2 min, and 3 min. The operators maintained a compression frequency as close to 5 Hz as possible, with a PEO concentration of 2000 ppm and a microchannel diameter of 200 μm. The t-test revealed no statistically significant differences (p > 0.05) in platelet aggregation areas between male and female operators.

**Long-term performance test.**

To evaluate the operational stability of the PHOMF device, we conducted long-term cycling tests over 1000 repeated operation cycles. A stretching machine (UTM6102, Sum technology, Shenzhen) was used to achieve uniform feeding motion for this test. Over a 17 min span, a tensile testing machine was employed to subject the PHOMF device to 1000 consecutive actuation cycles at a frequency of 1 Hz. The PHOMF device was subjected to 1000 single loading-unloading cycles to evaluate its cyclic performance (Fig. S-8). Throughout this period, the device showed consistent performance. This clearly indicates that the PHOMF device can be steadily operated to handle biological particles for a long time.


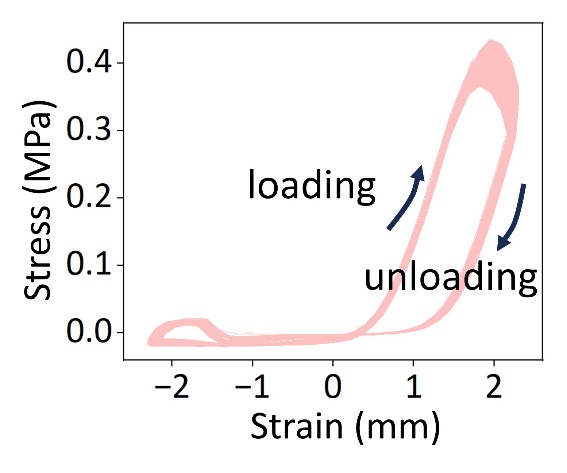


Fig. S-8 The stress-strain curve of the PHOMF device during tensile loading-unloading testing. The device was tested for 1000 cycles at an operating frequency of 1 Hz.

**Mixing effect.**

Oscillatory flow can significantly improve the fluid mixing.^[3]^ We utilize the PHOMF device to mix fluorescent dye and DI water to validate the mixing effect of oscillatory flow. 4 minutes later, the 0.2 M fluorescent dye is completely mixed with water (Fig S-9).


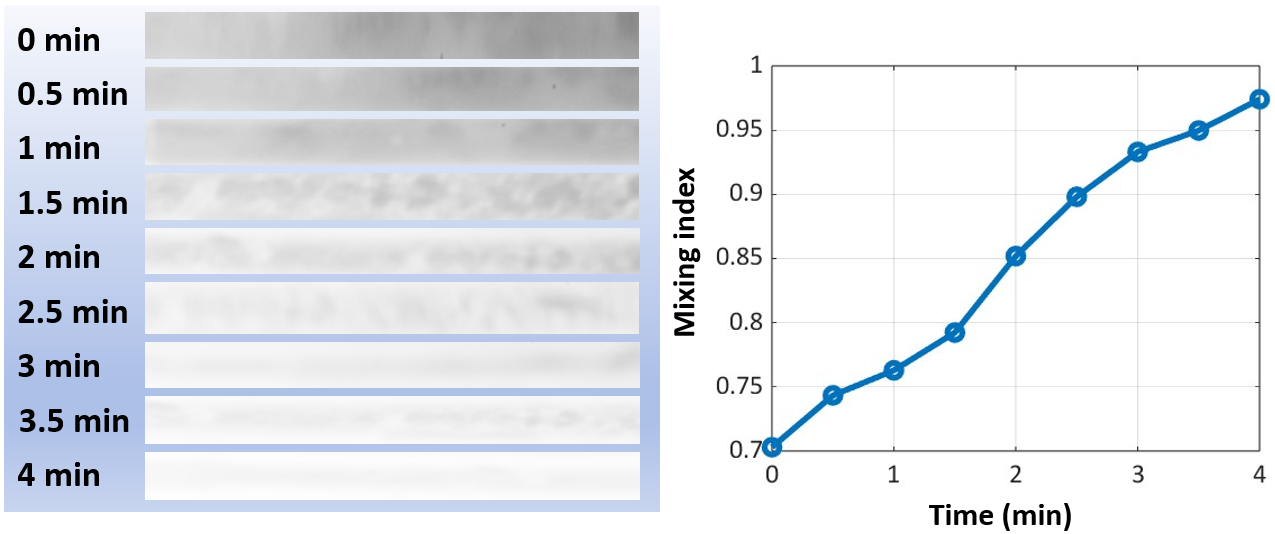


Fig. S-9 The mixing effect of the oscillatory flows in the PHOMF device is tested.

**Cell staining.**

To validate the PHOMF device for cell staining applications, we mixed 1 µL Membrane staining reagents (DiI, C1991S, Beyotime) with human triple-negative breast cancer cells (MDA-MB-231), infused the mixture into a 100 μm microchannel, and monitored the fluorescence intensity of cells during 480 s of PHOMF device operation at 5 Hz. For control experiments, MDA-MB-231 cells mixed with 1 µL Membrane staining reagents (DiI, C1991S, Beyotime) were transferred onto glass slides, and cell fluorescence was observed and captured using an inverted microscope. All cell suspensions were prepared in phosphate-buffered saline (PBS), a standard buffer solution for biological experiments. The fluorescence intensity of cells under PHOMF-generated oscillatory flow was significantly stronger than that under static conditions (Fig. S-10).


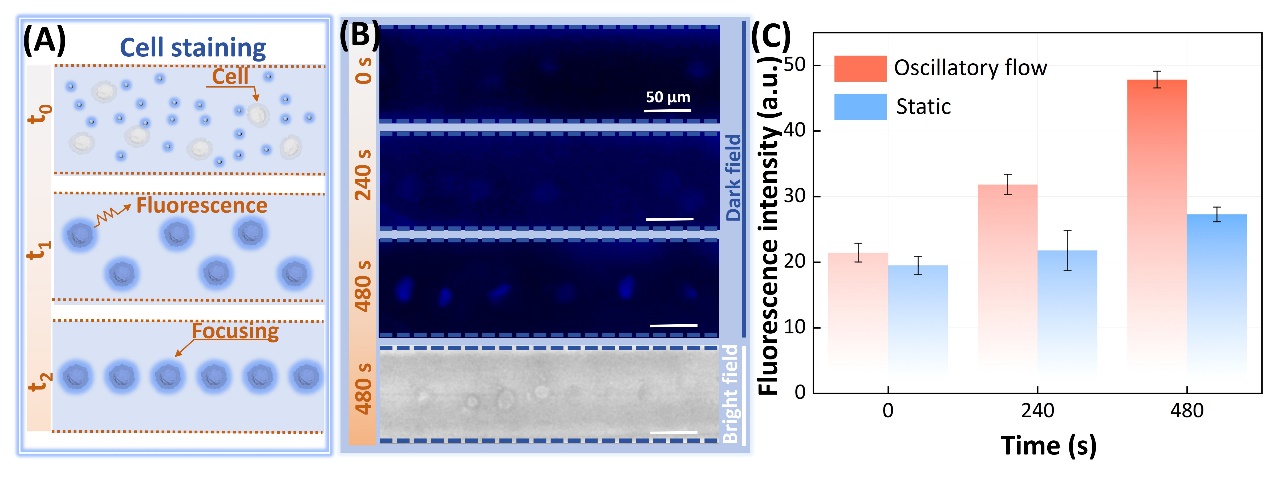


Fig. S-10 (A) Schematic diagram of cancer cells being dyed, emitting fluorescent signals and then being focused at the center of the microchannel. (B) The fluorescence and bright field images show the lateral positions of cancer cells in the microchannel and their fluorescence intensities within 480 s. (C) Comparison of cancer cell dyeing effects under oscillatory flow and at static state.

**Comparison of PHOMF devices with other technologies in particle/cell focusing, platelet aggregation, and cellular staining.**

To demonstrate the advantages of PHOMF devices in biological applications—including cell focusing, platelet aggregation, and cellular staining—we compared them with other devices and methods, as detailed in Tables S-1, S-2, and S-3. For particle/cell focusing, we compared the traditional mechanically-driven method with the pump-free control system method (Table S-1). This study employs a manually-driven approach to generate oscillatory flow fields for focusing particles and cells, offering the advantages of portability, miniaturization, and simplicity. Compared to unidirectional flow, oscillatory flow can achieve an effectively infinite flow path within a finite-length channel. This enables particle focusing within a short channel, reducing the chip footprint and effectively lowering flow resistance. Compared to Newtonian fluids, viscoelastic fluids with added polymers (e.g., PEO) enable both elastic lift forces and inertial lift forces to act on particles simultaneously. This not only reduces the required focusing distance but also shortens the necessary physical channel length.

The hydraulically amplified oscillatory microfluidic device we proposed requires a shorter microchannel length (L<3 mm) and operates at a lower particle Reynolds number (Re_p_<O(10⁻⁶)) compared to traditional unidirectional-flow microfluidic devices and other oscillatory microfluidic devices. However, this comes at the cost of lower throughput for biological particles in processed samples. By parallelizing these microchannels in the hydraulically amplified oscillatory microfluidic device, the throughput can be increased multiplicatively.

Platelet coagulation assays evaluate the performance of testing platforms by measuring the reaction time of coagulation in response to varying concentrations of agonists or anticoagulants (Table S-2). The oscillatory flow-induced shear stress in the PHOMF system enhances platelet activation via mechanical agitation. Moreover, the mixing effect of oscillatory flow enhances interplatelet interactions and facilitates contact between platelets and agonists/anticoagulants. For other microfluidic platforms, such as droplet-based systems, the droplet generation process and the pre-incubation of platelets with agonists/anticoagulants can significantly prolong the assay time. Compared to traditional methods, our hydraulically amplified microfluidic platform achieves faster platelet coagulation assays (t=3 min) while requiring lower concentrations of agonists/anticoagulants (c_ant._= 5 µM, c_ago._= 18 µM).

Cell staining techniques utilize fluorescent dyes that specifically bind to cellular components, enhancing contrast and enabling the detection of cell viability. Both excessive staining time and high fluorescent dye concentrations are detrimental to cell viability. Table S-3 summarizes and compares the required fluorescent dye concentrations and staining durations for cell staining assays across different platforms. Our proposed hydraulically amplified oscillatory microfluidic platform enhances cell staining efficiency by introducing mixing effects and controlled shear stress, which promote dye penetration into cells. This innovation significantly reduces both experimental time (8 min) and required fluorescent dye concentrations (1 µL).

Table S-1. Comparison of particle/cell manipulation under different driving methods.

| Flow field | Dimensions | Fluids | Particle Size | Flow rate | Applications | Ref. |
| --- | --- | --- | --- | --- | --- | --- |
| Unidirectional flow | Rectangular straight L=4, 1.5 mm | HA | 10 μm, 2 μm | 400 μL/min | *malaria parasites* + WBCs cascaded | ^[4]^ |
|  | Rectangular straight L=45 mm | PEO  +PBS | 15 μm, 10 μm, 5 μm | Q=9 mL/h | Rare tumour cells, Whole blood | ^[5]^ |
|  | Rectangular straight L=40 mm | PVP | 6 μm | Q=0.16, 0.24 mL/h | stiffen RBCs, health RBCs | ^[6]^ |
|  | Rectangular straight  L=30 mm | PEO | 3 μm, 1 μm | Q=0.1 mL/h | *E.coli*, RBCs | ^[7]^ |
|  | Circular straight  L=28 mm | PEO | 38 μm, 20 μm, 15 μm, 10 μm, 5 μm | Re=2.5 | Different sizes *Haematococcus pluvialis* | ^[8]^ |
|  | Circular straight  L=28 mm | PEO | 30 μm, 26 μm, 20 μm, 15 μm, 10 μm, 5 μm | Re=1.7, 3.5 | 3T3 cell, yeast cells | ^[9]^ |
|  | Rectangular straight  L=25 mm | PEO+PBS | 5 μm, 3 μm | Q=390 μL/h | *E.coli*, whole blood | ^[10]^ |
|  | Rectangular straight  L=15 mm | PEO+PBS | 2 μm,1 μm | Q=0.3 mL/h | *Staphylococcus aureus,* Platelets | ^[11]^ |
|  | Rectangular straight  L=15 mm | PEO | 3.2 μm, 2.5 μm, 3.2 μm | Q=60 μL/min | *E.coli + Platelet* | ^[12]^ |
|  | Rectangular straight  L=7 mm | Newtonian fluid+ PEO | 10 μm, 7 μm, 2 μm | Q=21.5 μL/min | *Alternaria alternata, Aspergillus niger NIH3T3 fibroblast cell, Cladosporium cladosporioides* | ^[13]^ |
|  | Rectangular straight L=5 cm, Rectangular spiral L=50 cm | HA+PBS | 10.2 μm, 6.27 μm, 3 μm | Q=500 μL/min | *Chlorella vulgaris* | ^[14]^ |
|  | Rectangular spiral L=13 cm | Newtonian fluid | 10 μm, 8 μm, 1 μm | Q=2 mL/min | None | ^[15]^ |
|  | Rectangular double spiral microchannel L=4 mm | PEO+Tris-EDTA | 2 μm, 100 nm | Re=0.61 | λ-DNA | ^[16]^ |
|  | Rectangular Spiral L>90 cm | Newtonian fluid | 15 μm, 10 μm, 7 μm | Q=500 μL/min | Plasma extraction | ^[17]^ |
|  | Rectangular straight L>2 cm | PEO | 10 μm, 5 μm, 3 μm | Q=50-500 μL/h | None | ^[18]^ |
| Oscillatory flow | Rectangular straight L=4 mm | PEO+PBS | 10 μm, 5 μm, 1 μm, 500 nm, 200 nm, 100 nm | Re = 0.03 -0.08 | λ-DNA, small extracellular vesicles | ^[19]^ |
|  | Rectangular straight L=40 mm | Newtonian fluid | 500 nm | Re_p_ < 0.005 | Staphylococcus aureus | ^[20]^ |
|  | **Circular straight L<3 mm (this paper)** | **PEO+ PBS** | **25 μm, 15 μm, 15 μm, 500 nm** | **Re_p_ < O (10^-6^)** | **PC12 cells, SW620 cells, MDA-MB-231 cells** |  |

Table S-2. Comparison of platelet coagulation and pharmacotherapy assays in the PHOMF platform with other platforms.

| Platform | Agonist (ago.)/ Anticoagulant (ant.) type | Agonist/ Anticoagulant concentration | Timing of the clotting | Ref. |
| --- | --- | --- | --- | --- |
| Printed circuit board-based digital microfluidics | Kaolin (ago.) | 5 mg/mL | 20 min | ^[21]^ |
|  | Heparin (ant.) | 8 U/mL |  |  |
| Droplet microfluidic platform (NebulaPlate) | Aspirin (ant.) | 100 µM | 40 min | ^[22]^ |
|  | Ticagrelor (ant.) | 30 µM |  |  |
| Droplet microfluidics | Calcium chloride (ago.) | 0.2 M | 12-15 min | ^[23]^ |
| Gravity-driven flow | Oligonucleotide (ago.) | 100 µM | 38 min | ^[24]^ |
|  | RPT-060318 (SYK inhibitor) (ant.) | 30 µM |  |  |
| **PHOMF (this paper)** | **Ticagrelor (ant.)** | **5 µM** | **3 min** |  |
|  | **ADP (ago.)** | **18 µM** |  |  |

Table S-3. Comparison of cellular staining capabilities between PHOMF devices and other technologies.

| Protocol | | Type of dye | Dye volume or concentration | Eefficiency or time | Target | Ref. |
| --- | --- | --- | --- | --- | --- | --- |
| Test tube | Well plate | Calcein AM, Propidium Iodide | 300 µL | Staining time: 30 min | Botrytis cinerea, Escherichia coli, Staphylococcus aureus, L929 mouse fibroblast cells | ^[25]^ |
|  | Well plate/petri dish | NucBlue | 1 mL | Staining time: 3 hours | Nucleus | ^[26]^ |
|  |  | TMRE |  | Staining time: 1 hours | Mitochondria |  |
|  | Well plate (nanostraw-electroporation system) | GFP, mCherry | 500 ng, 1500 ng | Eefficiency: >95% | human-induced pluripotent stem cell (iPSC)-derived cardiomyocytes, human embryonic stem cells, human fibroblasts, mouse glia cells, and mouse primary neuron cells | ^[27]^ |
| Microfluidics | Electroporation+shear effect | Dextran molecules | 0.2 mg/ml | Eefficiency: >70% | Breast cancer cells | ^[28]^ |
|  |  | Calcein AM | 50 µg/ml | Eefficiency: >70% | K562 and MDA-MB-231 cells | ^[29]^ |
|  | Open-close switchable microfluidic device | Trypan blue, Calcein-AM/PI | 5 µL | Staining time: 8 min | DU 145 cell spheroids | ^[30]^ |
|  | Super-resolution immunofluorescence staining by microfluidics | Annexin/PI | 5 µL | Staining time: 15 min | chronic myeloid leukemia derived cell line | ^[31]^ |
|  | **PHOMF (this paper)** | **DiI** | **1 µL** | **8 min** | **Breast cancer cells** |  |

**References.**

[1] G. D’Avino, G. Romeo, M. M. Villone, F. Greco, P. A. Netti, P. L. Maffettone, *Lab. Chip* **2012**, *12*, 1638.

[2] J. Zhou, I. Papautsky, *Microsyst. Nanoeng.* **2020**, *6*, 113.

[3] A. Mudugamuwa, U. Roshan, S. Hettiarachchi, H. Cha, H. Musharaf, X. Kang, Q. T. Trinh, H. M. Xia, N. Nguyen, J. Zhang, *Small* **2024**, *20*, 2404685.

[4] J. Nam, Y. Shin, J. K. S. Tan, Y. B. Lim, C. T. Lim, S. Kim, *Lab. Chip* **2016**, *16*, 2086.

[5] F. Tian, L. Cai, J. Chang, S. Li, C. Liu, T. Li, J. Sun, *Lab. Chip* **2018**, *18*, 3436.

[6] S. Yang, S. S. Lee, S. W. Ahn, K. Kang, W. Shim, G. Lee, K. Hyun, J. M. Kim, *Soft Matter* **2012**, *8*, 5011.

[7] C. Liu, C. Xue, X. Chen, L. Shan, Y. Tian, G. Hu, *Anal. Chem.* **2015**, *87*, 6041.

[8] Z. Jia, J. Wu, X. Wu, Q. Yuan, Y. Chan, B. Liu, J. Zhang, S. Yan, *Anal. Chem.* **2023**, *95*, 13338.

[9] P. Liu, Z. Jia, Y. Liu, S. Xu, X. Liu, R. Peng, S. Yan, *Phys. Fluids* **2024**, *36*, 092018.

[10] M. A. Faridi, H. Ramachandraiah, I. Banerjee, S. Ardabili, S. Zelenin, A. Russom, *J. Nanobiotechnology* **2017**, *15*, 3.

[11] F. Tian, W. Zhang, L. Cai, S. Li, G. Hu, Y. Cong, C. Liu, T. Li, J. Sun, *Lab. Chip* **2017**, *17*, 3078.

[12] H. Jeon, S. H. Lee, J. Shin, K. Song, N. Ahn, J. Park, *Microsyst. Nanoeng.* **2024**, *10*, 15.

[13] B. S. Park, H. G. Kye, T. H. Kim, J. M. Lee, C. D. Ahrberg, E.-M. Cho, S. I. Yang, B. G. Chung, *The Analyst* **2019**, *144*, 4962.

[14] M. J. Kim, J. R. Youn, Y. S. Song, *Lab. Chip* **2018**, *18*, 1017.

[15] A. A. S. Bhagat, S. S. Kuntaegowdanahalli, I. Papautsky, *Lab. Chip* **2008**, *8*, 1906.

[16] C. Liu, B. Ding, C. Xue, Y. Tian, G. Hu, J. Sun, *Anal. Chem.* **2016**, *88*, 12547.

[17] S. Shen, X. Liu, K. Fan, H. Bai, X. Li, H. Li, *Anal. Chem.* **2024**, acs.analchem.4c01549.

[18] D. Li, X. Lu, X. Xuan, *Anal. Chem.* **2016**, *88*, 12303.

[19] M. Asghari, X. Cao, B. Mateescu, D. van Leeuwen, M. K. Aslan, S. Stavrakis, A. J. deMello, *ACS Nano* **2020**, *14*, 422.

[20] B. R. Mutlu, J. F. Edd, M. Toner, *Proc. Natl. Acad. Sci.* **2018**, *115*, 7682.

[21] D. Li, X. Liu, Y. Chai, J. Shan, Y. Xie, Y. Liang, S. Huang, W. Zheng, Z. Li, *Lab. Chip* **2022**, *22*, 709.

[22] Z. Jiang, M. Wei, J. Zhu, C. Wang, T. Zhang, W. Zhu, R. Zhang, K. Zhang, P. Zhang, Y. Lu, A. C. Y. Chang, Y. Liu, J. Zhang, *J. Nanobiotechnology* **2025**, *23*, 171.

[23] L. Chen, D. Li, X. Liu, Y. Xie, J. Shan, H. Huang, X. Yu, Y. Chen, W. Zheng, Z. Li, *ACS Sens.* **2022**, *7*, 2170.

[24] K. Harada, W. Wenlong, T. Shinozawa, *Sci. Rep.* **2024**, *14*, 14109.

[25] G. Chen, K. Wang, P. Chen, D. Cai, Y. Shao, R. Xia, C. Li, H. Wang, F. Ren, X. Cheng, Y. Yu, *Adv. Sci.* **2024**, *11*, 2400826.

[26] C. J. Harry, J. D. Hibshman, A. Damatac, P. L. Davidson, M. A. Estermann, M. Flores-Flores, C. M. Holmes, J. Lázaro, E.-A. Legere, J. Leyhr, S. B. Thendral, B. A. Vincent, B. Goldstein, *STAR Protoc.* **2024**, *5*, 103232.

[27] Y. Cao, H. Chen, R. Qiu, M. Hanna, E. Ma, M. Hjort, A. Zhang, R. S. Lewis, J. C. Wu, N. A. Melosh, *Sci. Adv.* **2018**, *4*, eaat8131.

[28] D. A. L. Vickers, S. C. Hur, *J. Vis. Exp.* **2014**, 51702.

[29] H. Yun, S. C. Hur, *CBMS.* **2012**, 127.

[30] K. Ning, J. Li, X. Yang, Y. Xie, R. Pan, W. Sun, L. Yu, *Microchem. J.* **2024**, *206*, 111458.

[31] N. Jaafari, A. A. Kojabad, R. M. Shabestari, M. Safa, *PLOS ONE* **2025**, *20*, e0315803.
